# A dyadic examination of self-determined sexual motives, need fulfillment, and relational outcomes among consensually non-monogamous partners

**DOI:** 10.1371/journal.pone.0247001

**Published:** 2021-02-16

**Authors:** Jessica Wood, Christopher Quinn-Nilas, Robin Milhausen, Serge Desmarais, Amy Muise, John Sakaluk

**Affiliations:** 1 Department of Psychology, University of Guelph, Guelph, Ontario, Canada; 2 Department of Psychology, York University, Toronto, Ontario, Canada; 3 Sex Information and Education Council of Canada, Toronto, Ontario, Canada; 4 Department of Psychology, University of Waterloo, Waterloo, Ontario, Canada; 5 Department of Family Relations and Applied Nutrition, Guelph, Ontario, Canada; 6 Department of Psychology, University of Victoria, Victoria, British Columbia, Canada; 7 Department of Psychology, Western University, London, Ontario, Canada; Ohio State University, UNITED STATES

## Abstract

Intimate and sexual relationships provide opportunity for emotional and sexual fulfillment. In consensually non-monogamous (CNM) relationships, needs are dispersed among multiple partners. Using Self-Determination Theory (SDT) and dyadic data from 56 CNM partnerships (112 individuals), we tested how sexual motives and need fulfillment were linked to relational outcomes. We drew from models of need fulfillment to explore how sexual motives with a *second* partner were associated with satisfaction in the primary relationship. In a cross-sectional and daily experience study we demonstrated that self-determined reasons for sex were positively associated with sexual satisfaction and indirectly linked through sexual need fulfillment. Self-determined reasons for sex predicted need fulfillment for both partners at a three-month follow up. The association between sexual motives and need fulfillment was stronger on days when participants engaged in sex with an additional partner, though this was not related to satisfaction in the primary relationship. Implications for need fulfillment are discussed.

## Introduction

Intimate and sexual relationships are central to psychological well-being and provide an opportunity for emotional intimacy, sexual fulfillment, and personal growth [1]. However, maintaining satisfying sexual and romantic connections with long-term partners is challenging, with many couples reporting declines in relationship and sexual satisfaction over time [2]. High expectations of what modern romantic relationships entail (e.g., the expectation to receive love, comfort, emotional and financial support, sexual excitement, etc.) may place pressure on partners, making need fulfillment challenging [1]. One strategy for managing need fulfillment is the dispersion of needs among multiple partners, often referred to as *consensual non-monogamy* (CNM; [1,3]).

Consensual non-monogamy is an umbrella term used to describe relationships in which all partners have agreed that additional sexual, romantic, and/or intimate relationships are permitted [4,5]. Although individuals who participate in CNM indicate that their relationships include a broad range of structures, philosophies, boundaries, and identities, the most common forms of CNM described in the academic literature include open, polyamorous, and swinging relationships [4,6]. Open relationships primarily involve agreements in which established partners have consented to some form of extra-dyadic sex (whether together or separately; [7,8]). For example, a significant body of research has documented the relational agreements and outcomes of gay and bisexual men in non-monogamous relationships [7,9–16]. Polyamory is often described as the involvement (or potential involvement) in multiple romantic and/or sexual relationships, while swinging is frequently defined as retaining the emotional commitment to one’s primary partner but engaging in sexual behaviour with additional partners, often within the same social environment [4,6,8,17].

Approximately 3–7% of individuals in North America report currently participating in CNM [6,18,19] and almost one in five people have been involved in a CNM relationship at some point in their lives [18,19]. Although CNM relationships tend to be stigmatized and perceived as less fulfilling or satisfying than monogamous ones [5,20], there is growing evidence that individuals in CNM relationships report similar or sometimes higher scores on indicators of relational well-being [7–10,12–15,21–23]. While previous work has focused on comparing relational outcomes among people in CNM and monogamous relationships [8,21–28] and examining how sexual agreements are associated with satisfaction and well-being [11,29–35], little work has examined the motivational processes that contribute to need fulfilment in CNM relationships.

CNM relationships are unique in that multiple partners may contribute to need fulfillment [3,36]. Further, CNM individuals may have different sexual motives with different partners. For example, sexual variety and the desire for novel experiences are commonly cited motives for engaging in CNM or opening up a previously monogamous relationship [9,37]. Gay men in relationships report that creating extra-dyadic sexual agreements with their partner made them feel more secure in their relationship while simultaneously supporting their sexual needs and increasing feelings of satisfaction [9]. In polyamorous relationships, primary partners are more likely to meet a person’s nurturance needs, such as comfort and support, while secondary partners are more likely to meet a person’s erotic needs, such passion and sexual intensity [36]. Further, need fulfillment in one partnership is associated with relational outcomes with a different partner [38]. Thus, it is possible that sexual motives with one partner in a CNM relationship could differentially impact need fulfillment and relational outcomes in another partnership. CNM relationships provide the opportunity to examine theoretical aspects of need fulfilment in which needs are dispersed among multiple individuals.

### Self-determination theory: Sexual need fulfillment and satisfaction in CNM relationships

Self-determination theory (SDT) provides a framework to understand the contexts in which sexual needs are fulfilled and relational well-being occurs in CNM relationships. In romantic relationships, self-determination refers to authentically endorsing one’s engagement in the partnership, without feeling pressured or coerced by internal or external forces (e.g., another person, feelings of guilt or shame [39]). SDT emphasizes the importance of innate psychological needs (i.e., the need for competence, autonomy, and relatedness) in enhancing well-being and distinguishes between motivations that are intrinsically derived versus those that result from external pressure [40,41]. Sexual motives are considered on a continuum from reasons that are more self-determined to less self-determined [42]. For example, a person may be intrinsically motivated to engage in sex because the activity itself is pleasurable (*personal intrinsic motivation*) or because they enjoy feeling close to a partner (*relational intrinsic motivation*^21^). Individuals may also engage in sex for extrinsic reasons that reflect volition and a person’s values such as having sex because one feels that it is a central component of romantic relationships. In such cases, a person is engaging in sex due to external influences (e.g., social norms or expectations) but is choosing to engage in the behaviour as it aligns with their personal values (referred to as *integrated-identified regulation* in the SDT literature [42]). At the other end of the self-determination spectrum are motivations that are less reflective of self-determined goals and are often driven by external rewards/punishments or to manage feelings of guilt, shame, or anxiety [41]. For example, a person may have sex in order to obtain material benefits or avoid conflict with a partner (referred to as *external regulation*) or to boost their self-confidence in order to make themselves feel more desirable (labeled *introjection* in SDT). In addition to the proposed range of volitional motives, SDT includes a motivational state that reflects unintentional behaviours (i.e., *amotivation* [39,40]). That is, a person may be forced or coerced to engage in sexual activity or engage in sex without self-involvement (i.e., going through the motions of sex with no idea why).

The incremental approach to SDT (i.e., motives that are less to more self-determined) can provide important information about need fulfillment and relational well-being in CNM partnerships. The theory suggests that psychological need fulfillment mediates the relationship between sexual motives and relational outcomes [23,42,43]. For example, engaging in sex because one feels that it is important to their personal or relational growth is proposed to enhance psychological need fulfillment and thus increase relational and sexual satisfaction. In contrast, if a person were to engage in sex to avoid conflict with a partner, it is less likely that they would feel fulfilled by the sexual interaction, thus decreasing relational well-being. Research supports these suppositions and has determined the importance of self-determined sexual motivation to need fulfillment and relational outcomes in both monogamous and CNM individuals [23,42,44,45].

However, CNM relationships are unique in that partners are able to have their sexual and emotional needs fulfilled by multiple individuals, and each person in the partnership has the knowledge that their partner(s) are also having their own needs fulfilled by others. SDT suggests that partner dynamics significantly impact psychological need fulfillment and relational outcomes [43,46]. For example, when a person is in a relationship where their autonomy is supported by their partner, they can authentically express themselves [47]. This outcome has also been described in the SDT literature as “growth motivation” [48]. Research has noted the importance of autonomy, authenticity, and personal growth in individuals’ reasons for engaging in CNM [37]. Further, it is possible that when individuals are able to engage authentically with additional sexual partners, they feel more sexually fulfilled and, in turn, more appreciative of their primary relationship(s). Indeed, research on CNM indicates that having one’s sexual needs fulfilled outside of a primary partnership can enhance relational outcomes with a primary partner [9,16,38]. Interviews with gay men suggest that engaging in casual sex with additional partners was viewed as a positive aspect of their established relationship [16]. Men reported that casual sexual was enjoyable, increased their self-confidence, and enhanced their primary relationship by bringing them closer to their partner. In another study, men reported being motivated to make open relationship agreements because it made sex between them and their primary partner more intimate [9]. In research with gay male couples, the two most common reasons for creating an open relationship included building trust and building honesty with one’s primary partner [29]. Such findings are supportive of an *additive model* of need fulfillment, where having sexual and emotional needs met by multiple partners can increase one’s overall well-being and enhance both (or all) partnered relationships [49].

Though some research supports the idea that additional sexual relationships can enhance current partnerships [16,36,38], other findings indicate that individuals engage in additional partnerships to compensate for a lack of need fulfillment in a current relationship (i.e., a *compensation model* [38,49]). For example, if one’s sexual needs are not being met in a primary partnership, engaging in sex with a new person may offer protective benefits to the primary relationship by ensuring that the person is still engaging in sex that makes them feel satisfied and fulfilled. In survey research with gay male couples, 66% of men in open partnerships reported that they engaged in an open relationship in order to “protect the relationship” [29]. Evidence for this model has been also demonstrated in qualitative research with gay men: participants noted that extra-dyadic casual sex was important given the sexual discrepancies between them and their primary partner [16]. Engaging in sex outside of the primary partnership allowed them to meet their sexual needs despite differing sexual preferences and drives. Similarly, in a qualitative study examining people’s motives for engaging in CNM, participants described discrepancies in sexual desire as a reason for engaging in CNM; they reported that CNM allowed them to manage differences in an established partnership while meeting differential needs [37].

A third possibility is presented in the literature; the *contrast model* suggests that having needs met by a second partner could threaten and diminish satisfaction with the primary partnership [49]. That is, additional partners may create instability within the primary partnership when a person becomes emotionally or sexually distant in response to their partner’s needs being met by another person [49]. Finally, research on polyamorous relationships supports an *independent* approach to need fulfilment; i.e., having one’s needs fulfilled in one partnership may not significantly impact the relational outcomes of another partnership as the relationships are independent of one another [49].

The limited research in this area has focused on how *general* need fulfillment in one relationship impacts another partnership [49]. To our knowledge, only one study [38] has focused specifically on testing the links between sexual need fulfillment and satisfaction among multiple, concurrent partners. In two cross-sectional surveys, individuals in CNM relationships who reported greater sexual need fulfillment within a primary partnership also indicated higher relationship and sexual satisfaction with an additional partner, suggesting evidence for the additive model [38]. Though, among women, greater sexual need fulfillment with a secondary partner was associated with lower satisfaction in the primary partnership, potentially indicating support for the contrast model. However, dyadic research is needed to further explicate how need fulfillment and relational processes occur within CNM relationships.

### The current research

Applying SDT to CNM relationships allows for the examination of unique theoretical questions about how sexual motivations and need fulfilment in one partnership are associated with relational outcomes in *another* relationship. Though the central tenets of SDT are proposed to work similarly across social and relational contexts [40], few scholars have applied an SDT perspective to romantic partnerships that fall outside of traditional monogamous pairings. Much of the motivational literature in this area has been conducted within a mononormative and heteronormative framework (i.e., assumptions about relationships that position relational quality/health within the confines of heterosexual and monogamous partnerships; [50,51]). This has significant implications for the way that self-determination, need fulfillment, and relational outcomes are constructed, which relationships are considered to be “healthy”, and how we think about motivations within the context of intimate partnerships. Previous research, primarily with gay male couples, has reported that motivations for different sexual agreements are associated with satisfaction with the agreement and the relationship [29,30,32–35]. However, this work has primarily looked at relational and sexual agreements and has often not examined sexual motivations specifically or studied the association between self-determined motivations and need fulfillment—concepts identified as central to relational well-being in SDT. Finally, the limited research applying SDT to the study of CNM relationships has focused on individual cross-sectional surveys, with participants reporting on only one of their partners [23]. Including information from, and about, multiple members of the relationship is critical to understand the relational dynamics of need fulfillment, how partners’ sexual motivations are related to one another, and whether sexual need fulfillment is an additive, contrasting, compensatory, or independent process. Examining how the associations between sexual motives, need fulfillment, and relational outcomes fluctuate over time can provide an indication of how, and whether, motives and need fulfillment contribute to the longer-term maintenance of CNM relationships.

Thus, the purpose of the current research was to extend previous findings in two key ways: 1) by examining the *dyadic* associations between sexual motivations, need fulfillment, and relational outcomes among CNM partners who report having at least one committed live-in (or “primary”) partner, thus providing important information about how partner dynamics shape relational outcomes, and 2) by investigating how sexual motives and need fulfillment with a *second* partner were associated with relational outcomes in the *first* (i.e., live-in/primary) relationship. Testing SDT models in relationships where committed partners have relationships that are external to the dyad (i.e., a second sexual partner) offers insight into competing models of need fulfillment.

**Fig 1:**
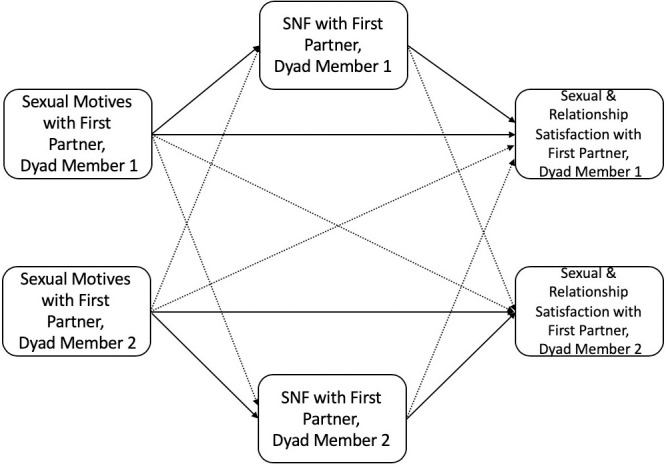
SDT model for sexual motives, sexual need fulfillment, and sexual/relationship satisfaction within the primary dyad. Notes: Solid lines indicate actor effects; dashed lines indicate partner effects; dyad members include CNM partners living with one another (often considered a “primary” partner, referred to as “first partner” in Fig 1); SNF = sexual need fulfillment.

**Fig 2:**
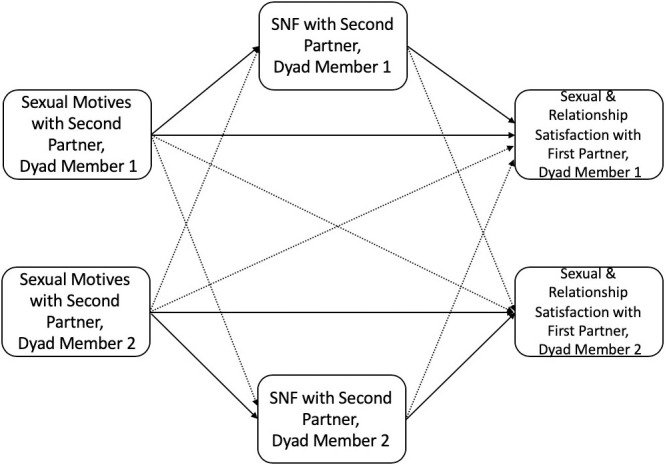
SDT model for sexual motives and sexual need fulfillment with a second partner, and sexual/relationship satisfaction in the primary dyad. Notes: Solid lines indicate actor effects; dashed lines indicate partner effects; dyad members include CNM partners living with one another (often considered a “primary” partner, referred to as “first partner” in Fig 2); “Second Partner” refers to another sexual partner that the person reported on who did not complete the study (i.e., is not a member of the dyad being analyzed); SNF = sexual need fulfillment.

We designed a two-part study (with the same participants) to test the SDT models in Figs 1 and 2. As participants are all from the same sample, we will refer to each part of the research as Part 1 and Part 2 for clarity. In Part 1, we examined cross-sectional data from an intake survey to test the indirect effect SDT models in Figs 1 and 2 (guided by the Actor-Partner Interdependence Model; APIM). In Part 2, we examined similar SDT models using dyadic diary data collected over the course of 21 days and tested whether sexual motives during this period were associated with relational outcomes at a three-month follow-up.

## Part 1

All participants reported on up to two partners. When referring to each partner that participants reported on, we have used the terms “first partner” and “second partner.” For ease of reading, we have also occasionally referred to the dyad as the “primary dyad.” Many members of CNM communities do not adhere to a hierarchical approach to relationships and the term “first partner” or “primary dyad” does not necessarily denote that this partner is “above” the second partner that the participants reported on. In all cases, the “first partner” refers to the person who completed the study with the participant (i.e., is a member of the dyad being analyzed and was a partner that they lived with) and “second partner” refers to a person who did not complete the study with the participant (i.e., is not a member of the dyad being analyzed).

Based on SDT and past research [23,42], we expected to replicate previous findings indicating that people who reported higher self-determined sexual motives also reported higher levels of sexual and relationship satisfaction. We further expected that this association would be indirectly linked through sexual need fulfillment. Consistent with dyadic research examining sexual motives and relational outcomes [44,52], we expected that a person’s own self-determined sexual motives would be positively linked to their first partner’s sexual and relationship satisfaction and that sexual need fulfillment would indirectly link these associations. To examine the different models of need fulfillment (i.e., additive, contrasting, compensation, or independent), we explored how sexual motives and sexual need fulfillment with a second, concurrent partner were linked to a person’s *own* relationship and sexual satisfaction (with a first partner) and the relationship and sexual satisfaction of their *first partner*.

## Methods

### Power analysis

Our goal was to recruit as large a sample as possible given that this is a hidden population that can be difficult to recruit. Power analyses were performed post hoc and are included here to provide context. Based on theorizing and results concerning SDT among non-CNM couples in the only other study to examine self-determined sexual motives and relational outcomes using an APIM model [31], we inferred a medium effect size of motives to relational outcomes for both actor (partial *r* = .52) and partner (partial *r* = .34) effects. To obtain these estimates, we pooled the effect sizes from Brunell and Webster [42] across their partial *r* reports for women and men. We used the APIMPOWER tool developed by Ackerman and colleagues [53] to estimate the sample size required for power (.80) given these details; 13 dyads were needed for the actor effects, and 30 for the partner effects (desired power = .80). Based on these analyses, we appear to have adequate power to detect both actor and partner effects. That said, there are limitations to using effect size estimates from a single prior study as the basis of power analysis; this could result in an underestimation of the sample size required. Furthermore, as indirect effects are typically of a smaller effect size, statistical power for the mediation models is likely lower. Without pilot data, there is no tool to estimate power for APIM using a daily diary methodology, however, given that longitudinal designs typically provide more statistical power than cross-sectional designs [54], we used the simple APIM power analysis as a base, but acknowledge that it is not a perfect representation of power for the daily diary component of this study.

### Participants

We recruited 61 partnerships (122 individuals) from online social media sites (e.g., Twitter), email listservs (e.g., polyweekly.com), and postings on reddit.com (e.g., r/polyamory). After one voluntary withdrawal from the study, and four dyads who were excluded because one or both partners did not complete the initial survey (this was required to proceed into the daily diary and subsequent follow-up), the final sample included 56 partnerships (112 individuals). Eligibility criteria included: 1) over the age of 18 and living in Canada or the United States, 2) fluent in English and have access to a computer, 3) currently in a CNM relationship with at least one committed partner, 4) currently living with at least one committed partner, 5) at least one of the two partners had to currently have at least one additional partner (in order to test cross-partner effects of having multiple concurrent relationships), 6) had sex with a partner at least once in the past month (to ensure that they could report on recent sexual events), 7) have one committed partner who was willing to participate in the study, 8) have a private email account that only they (and not their partner) had access to. Participants ranged in age from 19–65 years (*M* = 36.22, *SD* = 8.88). Length of relationship with the first partner ranged from six months to 26.92 years with an average of 8.88 years (*SD* = 6.91). This was significantly longer than relationship length with the second partner, *t*(96), = 4.32, *p* < .001, *d* = 1.29, which ranged from one month to 16 years with an average of 2.51 years (*SD* = 1.08). Frequency of partnered sexual interactions in the past month was significantly higher, *t*(101), = 8.70, *p* < .001, *d* = 0.61, with first partners (*M =* 9.28, *SD* = 10.06) compared to second partners (*M =* 4.33, *SD* = 5.60). Sixty-four percent (n = 34) of the dyads were in mixed-gender partnerships, 6% (n = 3) were in same-gender partnerships, and in 34% (n = 19) of the dyads at least one partner was gender queer, gender fluid, non-binary, or indicated multiple gender identities. There were 10 individuals who reported having no additional sex partner. See Table 1 for additional demographic information.

**Table 1:** Demographic characteristics.

Variable	N	%
**Gender**^**a**^		
Women (cisgender and transgender)	45	40.2
Men (cisgender and transgender)	43	38.4
Gender queer	12	10.7
Gender fluid	2	1.8
Non-binary	4	3.6
Multiple gender identities	1	0.9
**Sexual Orientation**		
Asexual	1	0.9
Lesbian	2	1.8
Gay	2	1.8
Bisexual	18	16.1
Pansexual	14	12.5
Queer	27	24.1
Uncertain or questioning	1	0.9
Heterosexual	39	34.8
Multiple orientations	2	1.8
Additional identities (e.g., heteroflexible, sapiosexual, demisexual)	6	5.4
**Racial/Ethnic Identities**^a^^b^		
Arab	1	0.9
Chinese	1	0.9
Multiracial	5	4.5
White	104	92.9
Choose not to answer	1	0.9
**Education**^**b**^		
Some high school	1	0.9
High school graduate	4	3.6
Some college/university	20	17.9
College/university graduate	37	33.0
Some trade/technical/vocational training	1	0.9
Trade/technical/vocational training degree or diploma	10	8.9
Some postgraduate work	7	6.3
Masters degree	20	17.9
Professional degree (e.g., MD)	7	6.3
Doctoral Degree	5	4.5
**Residence**		
Urban	66	58.9
Suburban	33	29.5
Rural	11	9.8
Other (e.g., small town)	2	1.8
**Relationship Type/Status**^a^^b^		
Open relationship (one or both of us has sex outside of the relationship)	12	10.7
Polyamorous (one or both of us are in multiple loving and/or sexual relationships)	45	40.2
Swinging relationship (one or both of you go to parties/clubs/etc. where partners may be exchanged for the night)	5	4.5
Multiple types of CNM	40	35.7
Other type of CNM (not specified)	10	8.9
Living with one partner	7	6.3
Living with multiple partners	1	0.9
Engaged to one partner	3	2.7
Engaged to more than one partner	3	2.7
Married to one partner	38	33.9
Married to more than one partner	3	2.7
Multiple types (e.g., married to one partner, casually dating another partner)	28	25.0

^a^Participants were asked to check all that apply.

^b^Additional categories were available but only those that were reported by participants are present in the table.

### Procedure

We pre-screened participants for eligibility via email and a phone call with the research team. During the phone call, all participants had the opportunity to go through the study components with the researcher and ask questions. Once both partners were enrolled, each received a link to an initial online survey, consisting of several demographic and relationship items, followed by questions related to sexual motives, sexual need satisfaction, relationship and sexual satisfaction, sexual desire, psychological well-being, interdependence, communication, communal strength, and overall motivations for engaging in CNM (see S1 File). Each participant answered relational and sexual questions for both a first partner and one second partner. Consent was obtained by participants clicking “yes” to the question “Do you consent to participate?”, after reading through the consent form. The intake survey took approximately 45 minutes to complete and participants were each paid $5.00. The Research Ethics Board at The University of Guelph cleared this research.

### Measures

#### Demographic questions

The demographic section included questions related to participants’ age, gender identity, ethnic and racial background, current geographical location, sexual orientation, relationship type/status, number of partners, and relationship duration.

#### Sexual motives

The 52-item Perceived Locus of Causality for Sex (PLOC-S [44]) assessed sexual motivations on a 5-point scale (*0 = not at all for this reason*, *4 = very much for this reason;* see Table 2 for Cronbach’s alpha scores). The stem for each question was “In the last month, I engaged in sexual activity with __” (partner’s initials inserted) and sample motives included: “Because I value sex as part of a full life,” “Because I thought sex would make me feel more secure,” and “But I don’t know why.” Subscales were: 1) *Personal Intrinsic Motivation* (8 items), 2) *Relational Intrinsic Motivation* (10 items), 3) *Integrated-Identified Regulation* (6 items), 4) *Introjected Regulation* (11 items), 5) *Extrinsic Regulation* (7 items), 6) *Amotivation* (4 items), and 7) *Drive Motivation* (6 items). Higher mean scores indicated higher levels of sex for each motivation subscale.

**Table 2:** Cronbach alphas, descriptive statistics and correlations for key variables for first partner and second partner at intake survey.

	First Partner			Second Partner										
Variable	α	M	SD	α	M	SD	1	2	3	4	5	6	7	8
1. Personal Intrinsic (+2)	0.90	5.63	1.76	0.89	6.17*	1.69	-	.61**	.68**	-0.15	.25**	.27**	0.05	.53**
2. Relational Intrinsic (+2)	0.89	5.84	1.51	0.91	6.00	1.65	.44**	-	.65**	-0.90	.25*	.36**	0.02	.52**
3. Integrated-Identified (+1)	0.87	2.86	0.93	0.91	2.83	1.04	.63**	.69**	-	-.23*	0.10	0.13	-0.15	.31**
4. Introjected (-1)	0.78	-0.70	0.55	0.78	-.82**	0.62	-.35**	-0.01	-.33**	-	.60**	.42**	.47**	0.16
5. Extrinsic (-2)	0.80	-0.37	0.81	0.83	-0.28	0.77	0.01	0.10	-0.01	.49**	-	.54**	.58**	.30**
6. Sexual Need Satisfaction	0.85	53.75	7.38	0.82	52.19	8.20	.24*	.58*	.34*	0.07	.31***	-	.55**	.61**
7. Relationship Satisfaction	0.90	6.33	0.87	0.85	5.69*	1.10	0.08	.49**	.26*	.27*	.29**	.62**	-	.23*
8. New Sexual Satisfaction Scale	0.88	47.34	7.68	-	-	-	-	-	-	-	-	-	-	-

Note. * *p <* .*05*,

** *p <* .*01*,

*** *p <* .*001* Correlations above the diagonal are for First Partner (i.e., the first partner reported on/primary partner) correlations below the diagonal are for Second Partner (i.e., the second partner that participants reported on).

Based on prior research using a SDT motivational framework [42,55,56], we created a weighted composite measure of *self-determined motives* by assigning weights to each of the PLOC-S subscales (except for the drive scale, which has not been included in previous weighted scales of self-determined motives; see Wood et al [23]). In the SDT literature, *personal intrinsic motives*, *relational intrinsic motives* and *integrated-identified motives* are considered more self-determined motives [40,43] and these scales were given weights of +2, +2, and +1, respectively. In contrast, *introjected regulation*, *extrinsic regulation* and *amotivation* are considered less self-determined forms of motivation and were assigned weights of -1, -2 and -2, respectively. However, given the lack of variation in item questions and the poor reliability of the *amotivation* subscale in the current study, this subscale was omitted from the composite measure (see below). The composite measure was then created by summing the scores across the weighted subscales. Overall levels of self-determined sexual motives were similar for participants reporting on their first partner and their second partner (M_first_partner_ = 13.46, SD_first_partner_ = 3.90; M_second_partner_ = 14.02, SD_second_partner_ = 3.68).

Reliability scores for the for the *amotivation subscale* were low for both the first (*a* = .56) and second partner (*a* = .61) with little variation in the items. When all subscale items were included, the Cronbach’s alpha score for the *extrinsic motives* subscale was *a* = .67 with the first partner and *a* = .33 with the second partner. Item-total statistics indicated that item 16 (“Because it helped me relax or get to sleep”) was problematic and was removed from the subscale. Once removed, reliability scores were *a* = .80 (first partner) and *a* = .83 motives (second partner).

#### Sexual need fulfillment

The degree to which participants experienced sexual need fulfillment with their first and second partners was assessed using the 9-item *Need Satisfaction Scale* [57]. All items began with “When I engage in sexual activity with _” (partner’s initials embedded) and included items such as *“…I am free to be who I am*,” and *“…*.*I feel loved and cared about*.*”* Response choices were rated on a 7-point scale (*1 = not at all true*, *7 = very true*).

#### Relationship satisfaction

Satisfaction with the first relationship was assessed with an adapted, shortened version of the *Quality of Marriage* scale [58,59]. Participants rated their agreement with six items on a 7-point scale, (1 *= very strong disagreement*, *7 = very strong agreement*). Sample items included “Right now my relationship with __ is strong” and “Right now I am unsure if my relationship with __ will last.” Items were averaged, with higher scores indicating greater relationship satisfaction.

#### Sexual satisfaction

The *New Sexual Satisfaction Scale- Short* (NSSS-S [60,61]) measured sexual satisfaction in the first partnership. Participants were asked to think about their sex life with their first partner over the past 6 months and rate their sexual satisfaction on 12 items such as “the quality of my orgasms” and “my partner’s sexual creativity” using a 5-point scale (*1 = not at all satisfied*, *5 = extremely satisfied*).

### Analytic approach

Descriptive statistics and bivariate correlations were calculated to provide an initial assessment of the data (see Table 2). Cronbach’s coefficient alphas were computed to examine the internal consistency of all scales and subscales.

#### SDT models

We analyzed the SDT models using multi-level modeling in SPSS v.25 [62], guided by the APIM [63]. Dyadic analyses with romantic partners are often treated as distinguishable (i.e., there is a systematic or meaningful way to order the two scores [63]) and much of the previous dyadic research on sexual motives has been conducted with heterosexual couples, using gender as the distinguishing variable. When there is not a systematic way to order the partners’ scores, the data is treated as indistinguishable (i.e., there is not a meaningful factor that defines dyad member 1 and dyad member 2). The decision to treat data as distinguishable (i.e., there is some meaningful factor to order the dyad [63]) is an important one. In the case of heterosexual couples, gender is often used as the distinguishing variable. However, our sample has several unique properties which make the application of a distinguishable variable difficult or impossible. It is important to note firstly that distinguishing variables must be meaningful and can be borne of both theoretical and empirical rationale–and arbitrary decisions must be avoided [63]. Our sample contains a substantial number (34%, *n* = 19) of dyads that contain at least one individual who did not report one of the binary gender options (e.g., nonbinary, agender, queer) and there are also several same-gender relationships (6%; *n* = 3). Our interest in this topic is not restricted to mixed-gender relationships and, indeed, a substantive goal of the recruitment proceedings was to collect this type of sample. Thus, distinguishing the dyads by gender was not a substantive goal of this study and, instead, the goal was to examine the effects in totality. Empirical tests of indistinguishability for our models were inconclusive, as there appeared to be no evidence of distinguishability at baseline among any of our models, and an inconsistent pattern of (in)distinguishability throughout our repeated measures (see S2 File for details of distinguishability tests). Given this inconsistency, we have included in the manuscript the indistinguishable versions of these analyses as they: 1) avoid excluding a significant proportion of our sample (39.29%); 2) are inclusive of our nonbinary participants and participants in a relationship with a partner of the same gender, who are often excluded from research [64]; and (3) avoid “Sin 1: Assuming that dyad members are distinguishable” [63, p.22]. However, for the analyses with evidence to support their distinguishability we also include tables summarizing those specific analyses in S2 File. Note that none of the gender interactions were statistically significant in these models, which indicated that the associations under investigation did not vary based on gender.

We tested the links between a person’s own sexual motives, sexual need fulfillment, and relationship and sexual satisfaction (referred to as actor effects), and the associations between a person’s own sexual motives, sexual need fulfillment, and their *first partner’s* relationship and sexual satisfaction (referred to as partner effects). We tested these associations first with reports within the primary dyad (i.e., sexual motives, need fulfillment and relational outcomes with one’s primary partner), and then examined the links with reports of the second partnership. That is, we tested whether sexual motives with a second partner, and sexual need fulfillment with a second partner, was linked to relational outcomes in the primary dyad. This allowed us to explore whether CNM partners experienced need fulfillment in their relationships as additive, contrasting, compensatory, or independent. We tested separate models for each outcome (and each IV) and all models included the reports of both partners in the dyad. The models were estimated as fixed effects. Statistical significance of each indirect effect was tested using the Monte Carlo method (20,000 repetitions) to estimate the 95% confidence intervals [65]. Relationship length with the primary partner was included as a predictor in each model in order to control for the effects of this variable (see S3 File for tables/results without controlling for this variable).

## Results

### Associations between sexual motives, sexual need fulfillment, and relationship/sexual satisfaction with the first partner

There were significant actor effects for sexual satisfaction, relationship satisfaction, and sexual need fulfillment (see Table 3). That is, when participants engaged in sex with their first partner for more self-determined reasons, they reported higher levels of sexual satisfaction, relationship satisfaction, and sexual need fulfillment. Additionally, when people felt more sexually fulfilled with their first partner, they reported higher levels of relationship and sexual satisfaction. A partner effect was also identified in that when participants engaged in sex more often for self-determined motives, their first partner also reported higher levels of sexual need fulfillment and relationship satisfaction. Relationship length was a significant positive predictor of relationship satisfaction but not for sexual satisfaction or sexual need fulfillment.

**Table 3:** Associations between actor and partner sexual motives and relationship satisfaction, sexual satisfaction, and sexual need fulfillment with the first partner.

	Relationship Satisfaction		Sexual Satisfaction		Sexual Need Fulfillment	
	*b* (SE)	*t*	*b* (SE)	*t*	*b* (SE)	*t*
Actor Motives	.05 (.02)	2.29*	1.01 (.16)	6.47***	.09 (.02)	5.00***
Partner Motives	.04 (.02)	2.01*	.14 (.15)	.94	.04 (.02)	2.28*
Relationship Length	.04 (.02)	2.18*	-.14 (.10)	-1.43	.01 (.01)	.47
Actor Sexual Need Fulfillment	.52 (.08)	6.19***	5.88 (.81)	7.30***	-----	-----
Partner Sexual Need Fulfillment	.14 (.08)	1.65	-.36 (.80)	-.46	-----	-----
Relationship Length	.03 (.01)	2.18*	-.20 (.09)	-2.14	-----	-----

Note: *b* values are unstandardized coefficients.

*p < .05,

** *p* < .01,

*** *p* < .001.

We then tested whether sexual need fulfillment indirectly linked the association between self-determined sexual motives and sexual satisfaction. A significant indirect actor effect, 95% CI [.30, .81], indicated that when participants reported more self-determined reasons for engaging in sex with their first partner, they also reported higher levels of sexual need fulfillment with that partner, which in turn, was associated with greater sexual satisfaction. There was no significant indirect partner effect CI [-.10, .06]. We also tested whether sexual need fulfillment indirectly linked sexual motives and relationship satisfaction. There was a significant indirect actor effect CI [.03, .07]; when participants reported more self-determined reasons for engaging in sex with their first partner, they also reported higher levels of sexual need fulfilment with that partner which, in turn, was linked to higher levels of relationship satisfaction. No significant indirect partner effects CI [-.01, .02] were identified for relationship satisfaction.

### Associations between sexual motives with a second partner, sexual need fulfillment with a second partner, and relationship/sexual satisfaction with a first partner

There were no significant associations between participants’ own sexual motives with a second partner, and their own sexual need fulfillment, sexual satisfaction, or relationship satisfaction (i.e., no significant actor effects; see Table 4). However, significant partner effects were identified; feeling more fulfilled by a second partner was negatively associated with the first partner’s sexual satisfaction and relationship satisfaction. In other words, feeling more fulfilled by a second partner was not associated with the person’s *own* sexual and relationship satisfaction in their first relationship (i.e., an actor effect), but when the person reported higher sexual need fulfillment with a *second* partner, the *first partner* reported *lower* levels of sexual satisfaction and relationship satisfaction (i.e., a partner effect)—suggesting support for a contrast model of need fulfillment.

**Table 4:** Associations between actor and partner sexual motives and sexual need fulfillment with the second partner, and relationship satisfaction and sexual satisfaction with the first partner.

	Relationship Satisfaction		Sexual Satisfaction		Sexual Need Fulfillment	
	*b* (SE)	*t*	*b* (SE)	*t*	*b* (SE)	*t*
Actor Motives	.04 (.03)	1.31	.23 (.22)	1.06	.02 (.03)	.87
Partner Motives	.01 (.03)	.38	-.01 (.22)	-.04	.17 (.03)	.68
Relationship Length	.03 (.02)	1.28	-.16 (.16)	-.99	-.01 (.02)	-.27
Actor Sexual Need Fulfillment	-.05 (.15)	-.32	1.64 (.90)	1.82	-----	-----
Partner Sexual Need Fulfillment	-.32 (.15)	-2.12*	-2.29 (.90)	-2.55*	-----	-----
Relationship Length	.04 (.02)	1.76	-.15 (.16)	-.99		

Note: *b* values are unstandardized coefficients.

*p < .05,

** *p* < .01,

*** *p* < .001.

We tested the SDT mediation model to determine whether sexual need fulfillment indirectly linked self-determined sexual motives and sexual/relationship satisfaction. There were no significant indirect actor effects CI [-.05, .16] or partner effects CI [-.19, .08] for sexual satisfaction. Further, no significant indirect actor effects CI [-.01, .01] or partner effects CI [-.02, .01] for relationship satisfaction were identified.

## Part 2

To extend the results of Part 1, we conducted a 21-day dyadic daily experience study to test our hypotheses and examine daily changes in the associations between sexual motives, sexual need fulfillment, and relationship and sexual satisfaction. Based on previous research and the results of Part 1, we predicted that on days when participants reported engaging in sex for more self-determined reasons, they and their first partner would report higher levels of sexual need fulfillment, and in turn, higher sexual and relationship satisfaction. In order to gain insight into the different models of need fulfillment (i.e., additive, contrasting, compensation, independent), we also explored whether these associations would be conditional depending on upon who participants were having sex with (i.e., their first partner or another partner). Finally, we examined whether sexual motives over the course of the 21-day study predicted relational outcomes with a first partner at a three-month follow-up.

### Procedure

In the second phase of the research, participants completed a brief online experience survey each day for 21 days and received $2.00 for every survey completed. A follow-up survey was sent three months later for which participants were paid $10. Participants completed a total of 1824 entries, for an average of 16.29/21 entries per person. Both partners had to complete the initial survey (analyzed in Part 1) in order to receive the subsequent invitations for the daily diary component and follow-ups (there was no minimum threshold of daily diary entries that a participant had to complete to continue their participation). Three months later participants were invited to participate in the three-month follow-up, of which 88 (out of 112) individuals responded.

### Daily measures

Participants completed measures of sexual motives, relationship and sexual satisfaction, sexual need fulfillment, and an indication of which partnered sexual event they were reporting on (i.e., one that included their first partner or another partner). Short measures were included in order to decrease participant burden (see S1 File). Measures used at the three-month follow-up were the same as the measures used in Part 1 of the study.

#### Engagement in partnered sexual activity

Each day, participants were asked “Did you engage in sexual interaction with a partner today?” (yes/no). They were asked to specify which partner they had sex with and which sexual interaction they were reporting on with the following items: 1) “When you engaged in sexual activity today, was it with….” (response choices: my primary partner, partner 2, a partner not listed here, choose not to respond), and 2) “Which sexual encounter are you reporting on today?” (response choices: one with my primary partner, one with partner 2, a partner not listed here, choose not to respond). The latter question was used in the analyses and recoded as “first/primary partner” and “another partner.” Participants reported engaging in sex with a partner on a total of 718 days (31% of possible days; 39% of valid daily diary data entries); the number of days that people had sex ranged from 0 to 17. Four individuals reported no sex during the 21-day daily diary period. Of the days individuals reported engaging in sex with a partner, most (*n* = 484; 68%) were with their first partner and the remaining were with either their second partner or another partner (*n* = 229; 32%).

#### Sexual motives

A shortened version of the PLOC-S [44] evaluated daily sexual motives. This measure included 20 items from the original PLOC-S measure, and four subscales: 1) personal intrinsic (α *=* .89), 2) relational intrinsic (α *=* .93), 3) introjected α *a =* .79), and 4) extrinsic α *=* .80).

#### Relationship satisfaction

Daily satisfaction with the first relationship was assessed using the same 6-item measure utilized in the intake/follow-up surveys [58,59].

#### Sexual satisfaction

We assessed sexual satisfaction with a first partner with a single item: “Overall, how satisfied are you today with the sexual aspect of your relationship?” with response options ranging from *1 = not at all satisfied to 5 = extremely satisfied*.

#### Sexual need fulfillment

Sexual need fulfillment during the sexual interaction was measured by modifying the Need Satisfaction Scale [57] to fit a daily experience format (see Brunell & Webster [42]). Participants indicated how they felt during the sexual interaction with their partner on six items (choiceful, competent, connected to my partner, a lot of closeness and intimacy, my feelings and wishes were respected, inadequate) with response options ranging from *1 = strongly disagree to 7 = strongly agree* (*a =* .85).

### Analytic approach

Longitudinal APIM was used to estimate the actor and partner effects using the daily diary data using MLM in SPSS (V.25). We partitioned all level 1 predictors into their within- and between-person counterparts by person-mean centering and aggregating, respectively [66,67]. As a result, our findings represent within-person differences (while accounting for between person variation) such that coefficients shown in the daily diary analysis are representative of changes in the dependent variable for every one-unit deviation from the person’s own mean.

To test the conditional indirect effect of self-determined sexual motives predicting sexual/relationship satisfaction through changes in sexual need fulfillment, as moderated by partner type (i.e., first vs. another partner), we estimated conditional process APIM models of the proposed SDT mechanism. In addition to the testing of the interaction term, we then estimated simple effects for each indirect pathway by re-centring partner type (i.e., changing which sexual partner type was coded as 0 for the analysis), and tested each conditional indirect path using the Monte Carlo method (20,000 repetitions [65]).

## Results

### Daily associations

As reported in Table 5, there were significant actor effects for sexual satisfaction and sexual need fulfillment. That is, on days when participants reported higher self-determined sexual motives than they typically did across the 21-day time period, they reported higher levels of sexual satisfaction and sexual need fulfillment. A partner effect was also identified for both of these associations. In other words, on days when people had sex for more self-determined reasons than they typically did across the study, their *first partner* also reported higher levels of sexual satisfaction and sexual need fulfillment. No significant actor or partner effects were identified for relationship satisfaction. All models also controlled for relationship length (which was a significant predictor of sexual satisfaction and sexual need fulfillment in the motives models).

**Table 5:** Daily associations between actor and partner sexual motives and sexual need fulfillment and daily relationship satisfaction, sexual satisfaction, and sexual need fulfillment.

	Relationship Satisfaction		Sexual Satisfaction		Sexual Need Fulfillment	
	*b* (SE)	*t*	*b* (SE)	*t*	*b* (SE)	*t*
Actor Motives	.01 (.01)	1.26	.07 (.02)	4.68***	.13 (.01)	8.70***
Partner Motives	.00 (.01)	.36	.03 (.02)	1.99*	.03 (.01)	2.23*
Relationship Length	-.01 (.01)	.88	-.03 (.01)	-2.55*	-.02 (.01)	-2.44*
Actor Sexual Need Fulfillment	.06 (.04)	1.68	.28 (.06)	5.13***	-----	-----
Partner Sexual Need Fulfillment	.00 (.04)	.09	.06 (.06)	1.01	-----	-----
Relationship Length	.01 (.01)	1.79	-.02 (.01)	-1.82	-----	-----

Note: *b* values are unstandardized coefficients; degrees of freedom ranged from 396.52 to 428.25.

*p < .05,

** *p* < .01,

*** *p* < .001.

### SDT indirect effects models

Next, we tested whether sexual need fulfillment indirectly linked the associations between self-determined sexual motives and sexual satisfaction. A significant indirect actor effect, 95% CI [.02, .05], indicated that on days when participants reported having sex for more self-determined reasons, they also reported higher levels of sexual need fulfillment, which in turn, was linked to higher sexual satisfaction in their first relationship (see Table 5). Though we did not find significant direct effects of sexual motives for relationship satisfaction, we wanted to test whether there was an indirect effect through sexual need fulfillment. There was no significant indirect actor effect of sexual motives on relationship satisfaction, CI [-.001, .02]. Further, no significant indirect partner effects were identified for either sexual satisfaction; CI [-.002, .007] or relationship satisfaction; CI [-.002, .003].

### Conditional process models: The role of sex partner

We conducted additional analyses to determine whether the direct and indirect effects of daily self-determined sexual motives on sexual satisfaction were conditional upon who participants were having sex with (i.e., their first partner or another partner). These analyses allowed us to examine whether engaging in sex for self-determined motives with *another* partner (i.e., a partner outside of the first/primary relationship) contributed to a person’s reports of sexual need fulfillment on the days that they engaged in sex. It also allowed us to determine if having sex for self-determined reasons with another partner was associated with sexual satisfaction in the first relationship. Finally, we also tested whether having sex with another partner was related to the *first partner’s* reports of sexual need fulfillment and sexual satisfaction (i.e., partner effects). The analyses enabled us to explore whether there was support for an additive, compensatory, contrast, or independent models of need fulfillment. No significant interactions were identified for the partner effects, however there were several significant interactions for the actor effects (see Table 6).

**Table 6:** Daily associations between actor and partner sexual motives, sexual need fulfillment, and sexual satisfaction (with participants’ first partner) by partner type.

		Sexual Need Fulfillment		Sexual Satisfaction	
		*b* (SE)	*t*	*b* (SE)	*t*
First Partner	Actor Motives FP	.10 (.01)	7.72***	.09 (.02)	5.19***
	Partner Motives FP	.04 (.01)	2.73**	.01 (.02)	.82
	Relationship Length FP	-.00 (.00)	-.07	-.02 (.01)	-2.46*
	Actor Sexual Need Fulfillment FP	-----	-----	.34 (.07)	4.88***
	Partner Sexual Need Fulfillment FP	-----	-----	.01 (.07)	.89
	Relationship Length FP	-----	-----	-.02 (.01)	-1.87
Another Partner	Actor Motives AP	.18 (.02)	8.70***	.02 (.03)	.77
	Partner Motives AP	.01 (.02)	.31	.08 (.03)	2.68**
	Relationship Length AP	-.00 (.00)	-.07	-.02 (.01)	-2.46*
	Actor Sexual Need Fulfillment AP	-----	-----	.08 (.10)	.81
	Partner Sexual Need Fulfillment AP	-----	-----	.13 (.10)	1.24
	Relationship Length AP	-----	-----	-.02 (.01)	-1.87

Note: *b* values are unstandardized coefficients.

*p < .05,

** *p* < .01,

*** *p* < .001.

FP: First partner.

AP: Another partner.

For the association between sexual motives and sexual satisfaction, there was a significant interaction between actor sexual motives and partner type (*b* = 07, SE = .03, *df* = 426.02, *t* = 2.09, *p* = .04, 95% CI [.004, .14]). That is, the direct positive link between self-determined sexual motives and sexual satisfaction was significant only when participants were reporting on their motives with their *first partner*. There was no direct link between self-determined sexual motives and sexual satisfaction (in the first relationship) on days when participants were having sex with another partner. That is, engaging in sex for self-determined motives with another partner was not significantly associated with a person’s sexual satisfaction in their first relationship.

There was also a significant interaction between actor sexual motives and partner type for the link between sexual motives and need fulfillment (*b* = -.08, SE = .02, *df* = 453.09, *t* = -3.26, *p* = .001, 95% CI [-.13, -.03]). On days when participants had sex with either a first partner or another partner for more self-determined reasons than they typically did across the study, they reported higher levels of sexual need fulfillment. However, this link was significantly stronger when participants reported having sex with *another partner*. That is, self-determined sexual motives appeared to contribute more to a person’s sexual need fulfilment on days when participants were having sex with another partner (i.e., a second partner)—suggesting an additive effect on one’s sexual need fulfillment.

Finally, there was a significant interaction between actor sexual need fulfillment and partner type for the association between sexual need fulfillment and sexual satisfaction (*b* = -.26, SE = .12, *df* = 430.85, *t* = -2.10, *p* = .04, 95% CI [-.50, -.02]). That is, the link between higher levels of sexual need fulfillment and sexual satisfaction was only significant for days when participants reported having sex with their first partner. In other words, on days when participants reported higher levels of sexual need fulfillment with their first partner than they typically did across the study, they also reported higher levels of sexual satisfaction with their first partner. However, feeling higher levels of need sexual fulfillment with another partner was not significantly related to sexual satisfaction in the first relationship—indicating that participants may perceive their relationships as independent from one another.

Next, we tested whether sexual need fulfillment indirectly linked the actor effects between self-determined sexual motives and sexual satisfaction for both a first partner and another partner. A significant indirect actor effect, 95% CI [.02, .05], indicated that on days when participants reported having sex for more self-determined reasons with a first partner, they also reported higher levels of sexual need fulfillment with their first partner, which in turn, was linked to higher sexual satisfaction with their first partner. There was no indirect actor effect of sexual motives with another partner on sexual satisfaction in the first relationship, 95% CI [-.01, .01]. That is, having sex for more self-determined reasons with another partner was positively associated with a person’s own sexual need fulfillment, but this did not translate into higher (or lower) levels of sexual satisfaction with the *first* partner (i.e., support for an independent approach to need fulfillment).

### Longitudinal effects of sexual motives on relationship satisfaction, sexual satisfaction, and sexual need fulfillment

Finally, we examined whether there were longitudinal effects of pursuing sex for more self-determined reasons. In this analysis, we used aggregate measures of both partners’ goals for sex over the course of the 21-day daily diary to predict relationship satisfaction, sexual satisfaction, and sexual need fulfillment with the first partner at a three-month follow-up. As noted in Table 7, the more participants reported engaging in sex with their partners for self-determined reasons over the course of the 21-day study, the higher their relationship satisfaction, sexual satisfaction, and sexual need fulfillment (with their first partner) was at the three-month mark. Further, when people engaged in sex for more self-determined reasons over the course of the study, their first partner reported higher levels of sexual need fulfillment at follow-up.

**Table 7:** Sexual motives predicting relationship satisfaction, sexual satisfaction, and sexual need fulfillment at a three-month follow-up.

	Relationship Satisfaction		Sexual Satisfaction		Sexual Need Fulfillment	
	*b* (SE)	*t*	*b* (SE)	*t*	*b* (SE)	*t*
Actor Motives	.33 (.11)	3.06**	.06 (.03)	2.32*	.19 (.02)	11.50***
Relationship Length	.11 (.06)	1.86	-.02 (.02)	-1.48	-.01 (.01)	-1.04
Partner Motives	-.12 (.11)	-1.08	-.004 (.03)	-.15	.14 (.02)	6.97***
Relationship Length	.08 (.06)	1.45	-.03 (.02)	-1.64	-.01 (.01)	-1.42

Note: *b* values are unstandardized coefficients. All models also include relationship length as a covariate.

*p < .05,

** *p* < .01,

*** *p* < .001.

## Discussion

This research drew on SDT to determine how sexual motives were linked to sexual need fulfillment and relational outcomes in CNM partnerships. We tested SDT models of need fulfillment within committed CNM partners (i.e., the primary dyad), and examined how sexual motives and sexual need fulfillment with a second, concurrent partner was associated with relational outcomes in the first partnership. The findings have important implications for SDT and inform our understanding of established models of need fulfillment in romantic relationships (i.e., additive, compensatory, contrasting, independent).

### Sexual motives and need fulfillment in the primary dyad

Our results replicate previous research demonstrating that engaging in sex for self-determined motives has implications for both a person’s own sexual satisfaction and need fulfillment and that of their (first) partner [42]. In both the cross-sectional and daily diary components of the current research, reporting more self-determined reasons for sex was associated with higher levels of sexual need fulfillment, and in turn, greater sexual satisfaction (i.e., significant indirect actor effects). Though identified in the cross-sectional component only, self-determined reasons for sex was also indirectly linked to a person’s own relationship satisfaction in the primary partnership, through sexual need fulfillment. While the indirect partner effects were not significant, we determined that when individuals had sex for more self-determined motives, their first partner also reported higher levels of sexual need fulfillment and relationship satisfaction. Similar actor and partner effects have been identified in research with monogamous participants. In a study with heterosexual undergraduate couples, the relationship between men’s daily self-determined sexual motivation and relational quality was mediated by sexual need satisfaction [42]. Further, men’s self-determined sexual motivation positively impacted women’s daily relationship satisfaction. Other daily experience studies with monogamous couples indicate that on days when one partner engaged in sex for reasons related to intimacy or pleasure, the other partner reported higher levels of relational satisfaction [39]. Though research with gay and bisexual men in open relationships has not specifically addressed SDT concepts, qualitative work with gay male couples suggests a similar pattern of results [9]. For example, men described intrinsically motivated reasons for creating open relationship agreements with their primary partner; they reported that the sense of trust they developed in their partner as a result of their agreement elevated and strengthened their relationship [9]. In some cases, both members of the couple described feeling closer sexually and relationally as a result of making an open relationship agreement and engaging in extra-dyadic sexual experiences.

Our findings suggest that the central tenets of SDT work in a similar fashion among live-in/primary CNM partners as they do with people who are in monogamous relationships, and gay male couples. That is, when individuals engage in sex because they want to experience pleasure or enhance intimacy, the sexual interaction is more likely to meet their psychological needs and is positively linked not only to how *they* view their sexual and romantic partnership, but also to how their *first partner* views the relationship. Thus, it appears that the benefits of self-determined motives are not limited to one’s own relational well-being but extend to one’s first partner as well.

Our study is among the first to examine the longer-term effects of self-determined sexual motives. We identified that self-determined sexual motives were positively linked to a person’s own relationship satisfaction, sexual satisfaction, and need fulfillment at the three-month follow-up. Further, when people engaged in sex for more self-determined reasons over the course of the 21-day study, their first partner also reported higher levels of sexual need fulfillment three months later. This suggests that the positive effects of having sex for self-determined motives are not only present on the days that people are engaging in sex but that there are lasting relational benefits to engaging in sex for self-determined reasons.

### Sexual motives and need fulfilment with a concurrent sexual partner: First partner relational outcomes

The current research provides novel insight into how sexual motives and sexual need fulfillment in a different, concurrent partnership are linked to relational outcomes in the first relationship. In the cross-sectional study, feeling more sexually fulfilled with a second partner was negatively associated with the first partner’s sexual satisfaction and relationship satisfaction (i.e., a partner effect). At first glance, this appears in line with contrast models of need fulfillment in CNM relationships where being fulfilled in one partnership negatively impacts how a person feels about a different relationship [49]. Yet, no actor effects were identified in the sexual satisfaction or relationship satisfaction models. That is, feeling sexually fulfilled by a second partner was not associated with how the *person* felt about their first relationship, but rather how their *first partner* felt about their sexual and relationship satisfaction in the partnership. In polyamorous relationships, individuals report spending a greater amount of time on sexual activity with a secondary partner, compared to a primary partner [36]. It is possible that a discrepancy in how partners spend time with one another impacts how they feel about the partnership(s). For example, if one member of the primary dyad desires more time spent connecting through sexual activity with their first partner and is unable to, their satisfaction could be negatively impacted when they learn of their partner’s sexual time with another individual. Yet, in the current study participants reported engaging in sex more frequently with their first partner (compared to their second partner), suggesting that frequency of sex (or possibly, time spent on sex) alone does not account for this finding.

The concept of new relationship energy (NRE) may contextualize the partner effect identified in Part 1. NRE is described in the CNM literature as intense feelings of excitement at the beginning of a new partnership [68,69]. NRE with a novel partner can enhance current relationships when a partner brings that additional energy into the primary relationship [38,69]. However, negative consequences of NRE have also been noted [70]. For example, established long-term partners can feel “left out” when their partner is intensely experiencing NRE, or find that their partner manages their time differently during this process. In such cases, it is possible that being aware of a partner’s sexual need fulfillment with another person could negatively impact how sexually and relationally satisfied that person feels in the primary dyad.

Importantly, the partner effect with sexual satisfaction identified in Part 1 did not replicate in the daily experience study. In the diary study, no significant partner effects were identified (i.e., the first partner did not report lower levels of sexual satisfaction). Thus, the veracity of the partner effects observed in the cross-sectional component should be examined in future studies. In terms of the actor effects, the link between self-determined sexual motives and sexual need fulfillment was significant for days when participants had sex with their first partner, and when they were having sex with another partner. However, the association between motives and need fulfillment was *stronger* when participants reported on sexual interactions with another partner, indicating that self-determined sexual motives also contribute to sexual need fulfillment in participants’ additional relationships and are more strongly linked to a person’s sexual need fulfillment. This suggests that consensually engaging in sex with multiple partners (for self-determined reasons) has an additive affect for a person’s *own* sexual need fulfillment, even though it may not translate into higher satisfaction within the primary dyad.

Further, sexual need fulfillment indirectly linked the association between sexual motives and sexual satisfaction only on days when people were reporting on sexual interactions (and outcomes) with their first partner. The indirect effects model was not significant when participants reported on sexual interactions with a second partner and relational outcomes with their first partner. This implies that participants viewed their partnerships as separate, rather than mutually influential. That is, having sex for more self-determined reasons with a second partner, and feeling sexually fulfilled with a second partner, was not linked to how people evaluated sexual satisfaction in their first relationship (i.e., an independent approach to need fulfillment). Similar results have been identified in research with polyamorous individuals [49]. In a cross-sectional survey, polyamorous participants reported on their general need fulfillment with two current partners. Need fulfillment with one partner was negatively associated with relationship satisfaction in the other relationship, but the variance accounted for in these analyses was very low (less than 1%). Further, there were no significant associations between need fulfillment in one relationship and commitment to the other partner. These findings, together with those of the daily diary in the current research, contrast with social perceptions of CNM relationships as inherently less satisfying or healthy [6,8,21] and suggest that engaging in multiple relationships does not necessarily have a positive or negative impact on the interpersonal well-being of concurrent partnerships [49].

### Strengths, limitations, and future directions

The research extends previous findings by testing theory-driven ideas of how sexual motives are linked to sexual need fulfillment and relational outcomes in CNM relationships. Though an SDT approach has been applied to sexual relationships that are monogamous [42] or casual [71], the present research is among the first to examine this theoretical framework in a sample of partners where sexual needs are consensually dispersed (i.e., CNM). This research is also among the first to collect dyadic data from participants in CNM relationships and to report on relational outcomes in multiple partnerships for the same person. Including the contributions of each partner is central for understanding the interpersonal dynamics of need fulfillment [46]. As such, a dyadic analysis was an ideal framework for testing the components of SDT; it allowed us to explore how sexual motives and need fulfillment in one partnership were associated with relational outcomes in a separate, concurrent relationship.

Nonetheless, several limitations warrant discussion. Both parts of the research used measures adapted from previous studies of monogamous individuals/couples to assess sexual motives, need satisfaction, and relational outcomes (this may account for some of the low reliability scores identified in the sexual motives measures). Although some measures may be adapted for use among CNM partners, they are often not validated within this population, and likewise are presumed to be appropriate for relationships of all descriptions without any articulation of measurement boundary conditions. Adherents to SDT propose that the theoretical concepts are universal [46], however, it is possible that there are additional motives for sex among CNM individuals not included in the SDT measure that may impact relational outcomes (for example, reconnecting with a live-in partner after they have been away with another person or motives related to authenticity and bringing one’s whole self to their relationships- key aspects of motivations for engaging in CNM generally [37]). Deeper measurement-focused studies into these—and other—relationship measures suggest that those in CNM relationships may indeed construe relational concepts in a different fashion [28]. It is therefore critical moving forward that relationship researchers question their assumptions about the generalizability of their constructs and measures, and that when measurements models substantially differ, that future research on CNM establish measures specifically designed for people in CNM partnerships.

Further, participants only reported on two (rather than all) of their partners. It is possible that additional relationships differentially influence relational outcomes of a primary dyad. For example, a second long-term partner may have a different relational dynamic with the primary dyad (such as family integration) compared to a new additional partner where NRE is present. Though analytically challenging if done quantitatively, research that includes all members of the relationship could illuminate the specific contexts in which additional partnerships might positively or negatively influence one another. Qualitative research in this area could also determine the nuanced circumstances that result in instances where NRE is beneficial or detrimental to an established partnership.

It is important to stress that the current research reflects a specific type of CNM relationship (i.e., at least one live-in/committed partner) and is not representative of the vast relationship configurations found under the CNM umbrella. Research on CNM has been criticized for focusing primarily on relational structures that are akin to monogamy, thus reproducing hierarchical and monormative understandings of romantic partnerships [72]. Such reproductions reinforce the idea that the “the couple”, or in this case the “primary couple”, is the ultimate form of romantic stability and fulfillment [73]. The current research may contribute to this concern, given its focus on primary partnership outcomes. Nonetheless, many relationships are categorized by one committed partner and additional sexual relationships. For example, in one study 74% of the 667 participants identified with the term “primary partner” [37]. Further, SDT proposes that the associations between self-determination and relational outcomes will be similar across social contexts [40,43]. Given this proposition, it is likely that the links between sexual motives, need fulfillment, and relational outcomes would be similar for relationships where there are multiple committed partners (e.g., a polyamorous “quad”) or relationships that are non-hierarchical. However, cross-partner effects may look different in such relational structures. Individuals in polyamorous communities often discuss the term “compersion” to describe feelings that are the opposite of jealousy (i.e., a feeling of empathetic joy when a partner is interested in, or has experiences with, another partner [70,74]). It is possible that in polyamorous relationships (where there is a greater chance of having *multiple* committed/live-in partners) the cross-partner associations may look different. For example, sexual need fulfillment with one’s second partner (where someone may be experiencing compersion) could be positively linked to the sexual satisfaction of one’s first partner (i.e., an additive effect on need fulfillment [49]). Future research could explore these associations amongst CNM partners who explicitly state that they do not adhere to hierarchal understandings of relationships (compared to those with a primary emotional attachment such as in swinging partnerships or many forms of open relationships).

Similarly, future research could examine intersections between gender, sexual orientation, and CNM relationship type to determine whether the links identified in the current research are replicated or whether changes emerge based on varying identities and experiences. Though the components of SDT are thought to work similarly across social contexts, it is possible that there may be different norms and experiences related to these identities that impact how people conceptualize sexual motives, need fulfillment, and relational outcomes.

Additionally, participants in the study were almost all white. Thus, the findings are reflective of a particular social context and do not address issues of intersectionality related to CNM and racial and ethnic identities. Studies with smaller, non-representative samples of CNM individuals have concluded that the practice of CNM is more common among white individuals [75]. However, other research indicates that people of colour are equally as likely to engage in CNM as white people [75,76]. Differences in the language used to identify oneself as a CNM community member (e.g. “polyamorous” is more often associated with whiteness) versus examining the specific behaviours individuals engage in and/or the relational agreements that people have made, may account for such discrepancies [75,77]. In other words, individuals may engage in sexual activity with a person other than their primary partner (consensually) but not identify with the term “polyamorous” or “consensually non-monogamous.” Future research should explore different avenues for recruiting CNM individuals (e.g., large scale studies on romantic relationships that include questions about CNM, rather than targeting CNM individuals specifically), discern how different recruitment terms impact the characteristics of the sample, and adjust these terms to ensure CNM people of colour are included.

Further, some of the partner effects identified in the current research were small. This is in line with previous findings where actor effects are moderate (or large) and partner effects are much smaller [42]. However, it is also possible that our sample was too small to detect additional partner effects. Analyses with increased power could possibly strengthen the partner effects or identify additional partner effects. Future research with larger samples of CNM dyads would be beneficial to see if the current results are replicable and robust.

Although participants were followed over the course of 21 days in Part Two, the correlational nature of the data impedes definitive answers regarding the directionality of the associations between self-determined sexual motives, sexual need fulfillment, and relational outcomes. While theoretical models of SDT propose that sexual motives are linked to relational outcomes through sexual need fulfillment, it may be the case that when individuals feel their needs are fulfilled, they seek out partnered sexual activity for more self-determined reasons and this impacts their relational and sexual satisfaction. Longitudinal research in this area would play an important role in identifying the causal relations between these variables and modeling trajectories of motives and need fulfillment over time. A longitudinal approach could examine how partner dynamics shape relationships and whether there are long-term effects (beyond a three-month time period) of sexual motives on need fulfillment and relational outcomes.

### Implications and conclusions

The current research contributes to a growing body of work that demonstrates the unique benefits of applying a SDT perspective to understanding close relationships and sexual behaviour [23,42,45,46,59,71]. The study presented here suggests that self-determination is important for the personal and interpersonal development of intimate and sexual partnerships. The findings also demonstrate that, for many individuals, sex in intimate partnerships is one avenue for personal need fulfillment.

Our findings have implications both for intimate and sexual partners wishing to enhance their relationship(s) and clinicians working with CNM and monogamous individuals/couples. Promoting self-determined reasons for engaging in sex could encourage partners to engage in sexual interactions that are more likely to fulfill their needs (e.g., having sex when they are excited about the activity, rather than to avoid conflict). Encouraging partners to explore why they may be having sex for less self-determined reasons, and how they may shift to having sex for more self-determined reasons, is one strategy clinicians can use to promote relational well-being. Clinicians working with CNM partners can also encourage individuals to communicate and express continued affection and desire for established partners when new relationships occur in order to maintain sexual and relationship satisfaction in the primary dyad.

The current research also has implications for individuals in CNM communities. Popular assumptions of romantic relationships position CNM partnerships as less satisfying or less stable compared to monogamous relationships [6,20]. CNM partners in the current research noted high levels of satisfaction and sexual need fulfilment with both their first and second partners. Moreover, a concurrent sexual partnership did not appear to have significant detrimental effects on the first relationship. These findings verify what CNM researchers and advocates have previously emphasized: that for some, CNM relationships are a viable and fulfilling alternative to monogamy, and one of many approaches to encouraging personal growth and fulfillment [49]. These results may help to destigmatize CNM partnerships as they confirm that individuals can experience psychological need fulfillment and satisfying relationships with concurrent partners.

Finally, research on sexual behaviour, and on CNM generally, has been criticized for lacking theoretical frameworks [4,21,42,78]. The current studies contribute to a growing body of research that utilizes social psychological approaches to the study of sexual behaviour and emphasizes the importance of sexuality to relational well-being [36,42,45,52,71]. The research provides a theoretical context within which to understand the associations between sexual motives, need fulfilment, and relational outcomes in relationships where sexual and emotional needs are met by multiple partners, thus expanding the experiences represented in the social psychological literature.

## Supporting information

S1 File (PDF)

S2 File (DOCX)

S3 File (DOCX)

## References

[1] Finkel EJ, Hui CM, Carswell KL, Larson GM. The suffocation of marriage: Climbing mount Maslow without enough oxygen. Psychol Inq. 2014;25:1–41.

[2] McNulty JK, Wenner CA, Fisher TD. Longitudinal associations among relationship satisfaction, sexual satisfaction, and frequency of sex in early marriage. Arch Sex Behav. 2016;45:85–98. 10.1007/s10508-014-0444-6 25518817 PMC4472635

[3] Conley TD, Moors AC. More oxygen please! How polyamorous relationship strategies might oxygenate marriage. Psychol Inq. 2014;25:56–63.

[4] Barker M, Langdridge D. Whatever happened to non-monogamies? Critical reflections on recent research and theory. Sexualities, 2010;13(6):748–772.

[5] Conley TD, Moors AC, Matsick JL, Ziegler A. The fewer the merrier?: Assessing stigma surrounding consensually non-monogamous romantic relationships. Anal Soc Iss Pub Pol. 2012;13(1):1–30.

[6] Conley TD, Ziegler A, Moors AC, Matsick JL, Valentine B. A critical examination of popular assumptions about the benefits and outcomes of monogamous relationships. Pers Soc Psychol Rev. 2013;17(2):124–141. 10.1177/1088868312467087 23175520

[7] LaSala MC. (2004). Extradyadic sex and gay male couples: Comparing monogamous and nonmonogamous relationships. Fam Soc;405–412.

[8] Rubel AN, Bogaert AF. (2014). Consensual nonmonogamy: Psychological well-being and relationship quality correlates. J Sex Res. 2014;52(9):1–22. 10.1080/00224499.2014.942722 25189189

[9] Hoff CC, Beuogher SC. Sexual agreements among gay male couples. Arch Sex Behav. 2010;39:774–787. 10.1007/s10508-008-9393-2 18686027 PMC2855749

[10] Hosking W. Australian gay men’s satisfaction with sexual agreements: The roles of relationship quality, jealousy, and monogamy attitudes. Arch Sex Behav. 2014;43 823–832. 10.1007/s10508-013-0197-7 24287963

[11] Mitchell JW. Characteristics and allowed behaviors of gay male couples’ sexual agreements. J Sex Res. 2014;51:316–23. 10.1080/00224499.2012.727915 23514544 PMC4322899

[12] Parsons JT, Starks TJ, Gamarel KE, Grove C. Non-monogamy and sexual relationship quality among same-sex male couples. J Fam Psychol. 2012;26:669–677. 10.1037/a0029561 22906124

[13] Ramirez OM, Brown J. Attachement style, rules regarding sex, and couple satisfaction: A study of gay male couples. Aust. N. Z. J. Fam. Ther. 2010;31:202–213.

[14] Wagner GJ. Remien RHM, Carballo-Dieguez A. (2000). Prevalence of extradyadic sex in males couples of mixed HIV status and its relationship to psychological distress and relationship quality. J Homosex. 2000;39:31–46. 10.1300/J082v39n02_02 10933280

[15] Whitton SW, Weitbrecht EM, Kuryluk AD. Monogamy agreements in male same-sex couples: Associations with relationship quality and individual well-being. J Couple Relatsh Ther. 2015;14:39–63.

[16] Bonello K, Cross MC. Gay monogamy: I love you but I can’t have sex with only you. J Homosex. 2010;57:117–139. 10.1080/00918360903445962 20069497

[17] Jenks RJ. Swinging: A review of the literature. Arch Sex Behav. 1998;27:507–521. 10.1023/a:1018708730945 9795730

[18] Haupert M, Gesselman A, Moors A, Fisher H, Garcia J. Prevalence of experiences with consensual non-monogamous relationships: Findings from two nationally representative samples of single Americans. J Sex Marital Ther. 2017;20:1–17. 10.1080/0092623X.2016.1178675 27096488

[19] Fairbrother N, Hart T, Fairbrother M. Open relationship prevalence: Characteristics, and correlates in a nationally representative sample of Canadian adults. J Sex Res. 2019 56(6):695–704. 10.1080/00224499.2019.1580667 30932711

[20] Séguin LJ. The good, the bad, and the ugly: Lay attitudes and perceptions of polyamory. Sexualities, 2017;1363460717713382.

[21] Conley TD, Mastick JL, Moors AC, Ziegler A. Investigation of consensually nonmonogamous relationships: Theories, methods and new directions. Perspectives on Psychol Sci, 2017;12(2):205–232. 10.1177/1745691617710935 28346120

[22] Séguin LJ, Blais M, Goyer MF, Lavoie F, Rodrigue C, Magontier C. Examining relationship quality across three types of relationship agreements. Sexualities. 2016;20(1–2):1–19.

[23] Wood J, Desmarais S, Burleigh T, Milhausen RR. Reasons for sex and relational outcomes in consensually non-monogamous and monogamous relationships: A self-determination theory approach. J Soc Pers Relat. 2018; 35(18):632–654.

[24] Conley TD, Piemonte JL, Gusakova S, Rubin JD. Sexual satisfaction among individuals in monogamous and consensually non-monogamous relationships. J Soc Pers Relat. 2018; 34(4):509–531.

[25] Fleckenstein JR, Cox DW. (2014). The association of an open relationship orientation with health and happiness in a sample of older US adults. Sex & Relat Therapy. 2014;30: 94–116.

[26] Mogilski JK, Memering SL, Welling LLM, Shackelford TK. (2017). Monogamy versus consensual non-monogamy: Alter- native approaches to pursuing a strategically pluralistic mating strategy. Arch Sex Behav. 2017;46:407–417. 10.1007/s10508-015-0658-2 26679305

[27] Parsons JT, Starks TJ, Gamarel KE, Grov C. Non-monogamy and sexual relationship quality among same-sex male couples. Journal of Family Psychology. 2012;26(5):669 10.1037/a0029561 22906124

[28] Sakaluk JK, Quinn-Nilas C, Fisher AN, Leshner CE, Huber E, Wood J. Sameness and difference in psychological research on consensually non-monogamous relationships: The need for invariance and equivalence testing. Arch Sex Behav, In press; 10.1007/s10508-020-01794-9 32860096

[29] Hoff CC, Beougher SC, Chakravarty D, Darbes LA, Neilands TB. Relationship characteristics and motivations behind agreements among gay male couples: Differences by agreement type and couple serostatus. AIDS care. 2010;22(7):827–35. 10.1080/09540120903443384 20635246 PMC2906147

[30] Hosking W. Agreements about extra-dyadic sex in gay men’s relationships: Exploring differences in relationship quality by agreement type and rule-breaking behavior. Journal of Homosexuality. 2013;60(5):711–33. 10.1080/00918369.2013.773819 23593955

[31] Mitchell JW. Between and within couple-level factors associated with gay male couples’ investment in a sexual agreement. AIDS and Behavior. 2014;18(8):1454–65. 10.1007/s10461-013-0673-z 24327185 PMC4397649

[32] Mitchell JW, Harvey SM, Champeau D, Moskowitz DA, Seal DW. Relationship factors associated with gay male couples’ concordance on aspects of their sexual agreements: Establishment, type, and adherence. AIDS and Behavior. 2012;16(6):1560–9. 10.1007/s10461-011-0064-2 22012148 PMC4096805

[33] Rios-Spicer R, Darbes L, Hoff C, Sullivan PS, Stephenson R. Sexual agreements: a scoping review of measurement, prevalence and links to health outcomes. AIDS and Behavior. 2019;23(1):259–71. 10.1007/s10461-018-2212-4 29959719

[34] Mitchell JW, Champeau D, Harvey SM. Actor–partner effects of demographic and relationship factors associated with HIV risk within gay male couples. Archives of sexual behavior. 2013;42(7):1337–45. 10.1007/s10508-012-9985-8 22875716 PMC4388025

[35] Rogers E, Mimiaga MJ, Garofalo R, Brown E, Bratcher A, Wimbly T, et al A Dyadic Perspective on Sexual Agreements Among Same-Sex Male Couples in the United States. AIDS and behavior. 2020;4:3107–3123. 10.1007/s10461-020-02865-7 32300992 PMC11998888

[36] Balzarini RN, Dharma C, Muise A, Kohut T. Eroticism versus nurturance: How eroticism and nurturance differs in polyamorous and monogamous relationships. Social Psychology. 2019;50:185–200.

[37] Wood J., De Santis C, Milhausen R., & Desmarais S. Motivations for engaging in consensually non-monogamous relationships. Arch Sex Behav. In Press. 10.1007/s10508-020-01873-x 33990929

[38] Muise A., Laughton A., Moors A.C., & Impett E.A. (2018). Sexual need fulfillment and satisfaction in consensually non-monogamous relationships. J Soc Pers Relat. 2018;36(7):1917–1938.

[39] Knee CR, Hadden BW, Porter, Rodriquez LM. (2013). Self-determination theory and romantic relationship processes. Pers Soc Psychol Rev. 2013;17(4): 307–324. 10.1177/1088868313498000 23921674

[40] Deci EL, Ryan RM. The “what” and “why” of goal pursuits: Human needs and the self-determination of behavior. Psychol Inq. 2000;11:227–268.

[41] Ryan RM, Deci EL. Self-determination theory and the facilitation of intrinsic motivation, social development, and well-being. Am Psychol. 2000;55:68–78. 10.1037//0003-066x.55.1.68 11392867

[42] Brunell A, Webster G. Self-determination and sexual experience in dating relationships. Pers Soc Psychol B. 2013;39:970–987. 10.1177/0146167213485442 23613122

[43] Deci EL, Ryan RM. Self-determination theory In Van Lange AM, Kruglanski AW, Higgins ET, editors. Handbook of theories of social psychology: Volume 1 London: SAGE Publications, Ltd, 2012 P. 416–438.

[44] Jenkins SS. Gender and self-determination in sexual motivation. Dissertation Abstracts International: Section B: The Sciences and Engineering. 2004;64(12-B):6330.

[45] Smith V. In pursuit of “good” sex: Self-determination and the sexual experience. J Soc Pers Relat. 2007;24:69–85.

[46] LaGuardia JG, Patrick H. Self-determination theory as a fundamental theory of close relationships. Can Psychol. 2008;49:201–209.

[47] LaGuardia JG. On the role of psychological needs in healthy functioning: Integrating a self-determination theory perspective with traditional relationship theories In Wood JV, Tesser A, Holmes J, editors. Self and relationships. New York: Psychology Press; 2007.

[48] Knee CR, Patrick H, Vietor NA, Nanayakkara A, Neighbors C. (2002). Self-determination as growth motivation in romantic relationships. Pers Soc Psychol B. 2002;28(5):609–619.

[49] Mitchell ME, Bartholomew K, Cobb RJ. Need fulfillment in polyamorous relationships. J Sex Res. 2014;5:329–339. 10.1080/00224499.2012.742998 23541166

[50] Moors AC, Schechinger H. (2014). Understanding sexuality: Implications of Rubin for relationship research and clinical practice. Sex Rel Ther. 2014;29:476–482.

[51] Pieper, M. Bauer, R. Call for papers: International conference on polyamory and mono- normativity. University of Hamburg, 5–6 November 2005.

[52] Muise A, Impett EA, Desmarais S. Getting it on vs. getting it over with: Approach-avoidance sexual motivation, desire and satisfaction in intimate bonds. Personality and Social Psychology Bulletin. 2013;39:1320–1332. 10.1177/0146167213490963 23812928

[53] Ackerman, RA, Kenny, DA. APIMPower: An interactive tool for Actor-Partner Interdependence Model power analysis [Computer software]. (2016, December). Available from https://robert-a-ackerman.shinyapps.io/apimpower/.

[54] Guo Y, Logan HL, Glueck DH, Muller KE. Selecting a sample size for studies with repeated measures. BMC Med Res Methodol. 2016;13(1):100. 10.1186/1471-2288-13-100 PMC3734029 23902644

[55] Blais MR, Sabourin S, Boucher C, Vallerand RJ. Toward a motivational model of couple happiness. J Pers Soc Psychol. 1990;59(5):1021.

[56] Vallerand RJ, Pelletier LG, Koestner R. Reflections on self-determination theory. Can Psychol. 2008;49(3):257.

[57] La Guardia JG, Ryan RM, Couchman CE, Deci EL. Within-person variation insecurity of attachment: A self-determination theory perspective on attachment, need fulfillment, and well-being. J Pers Soc Psychol. 2000;79:367–384. 10.1037//0022-3514.79.3.367 10981840

[58] Norton R. Measuring marital quality: A critical look at the dependent variable. J Marriage Fam. 1983;45(1):141–151.

[59] Knee CR, Lonsbary C, Canevello A, Patrick H. Self-determination and conflict in romantic relationships. J Pers Soc Psychol. 2005;89(6):997 10.1037/0022-3514.89.6.997 16393030

[60] Fisher TD, Davis CM, Yarber WL, Davis SL. Handbook of Sexuality-Related Measures, 3rd Ed. New York: Routledge, 2011.

[61] Štulhofer A, Buško V, Brouillard P. Development and bicultural validation of the new sexual satisfaction scale. J Sex Res. 2010;47(4):257–268. 10.1080/00224490903100561 19629836

[62] IBM Corp. Released 2017. IBM SPSS Statistics for Windows, Version 25.0. Armonk, NY: IBM Corp.

[63] Kenny DA, Kashy DA, Cook WL. Dyadic data analysis. New York, NY: Guilford Press, 2006.

[64] Andersen JP, Zou C. Exclusion of sexual minority couples from research. Health Science Journal. 2015;9(6):1.

[65] Selig, JP, Preacher, KJ. Monte Carlo method for assessing mediation: An interactive tool for creating confidence intervals for indirect effects [Computer software]. (2008, June) Available from http://quantpsy.org/.

[66] Raudenbush SW, Bryk S, Cheong YF, Congdon R. HLM6: Hierarchical linear and nonlinear modeling. Chicago: Scientific Software International, 2004 10.1146/annurev.publhealth.25.050503.153925

[67] Zhang Z, Zyphur MJ, Preacher KJ. Testing multilevel mediation using hierarchical linear models: Problems and solutions. Organ Res Methods. 2009;12(4):695–719.

[68] Hardy JW, Easton D. The Ethical Slut. New York: Ten Speed Press, 2017.

[69] Wosick-Correa K. Agreements, rules and agentic fidelity in polyamorous relationships. Psychology & Sexuality. 2010;1:44–61.

[70] Taormino T. Opening Up: A Guide to Creating and Sustaining Open Relationships. San Francisco, CA: Cleis Press, 2008.

[71] Vrangalova Z. Does casual sex harm college students’ wellbeing? A longitudinal investigation of the role of motivation. Arch Sex Behav. 2014;44(4):945–959. 10.1007/s10508-013-0255-1 24496788

[72] Finn MD. Freedom in practices of non-monogamous commitment In: Barker M, Langdridge D, editors. Understanding non-monogamies. New York: Routledge; 2010.

[73] Finn MD, Malson H. Speaking of home truth: (Re)productions of dyadic-containment in non-monogamous relationships. Br J Soc Psychol. 2008;47:519–533. 10.1348/014466607X248921 17949533

[74] Ritchie A, Barker M. ’There aren’t words for what we do or how we feel so we have to make them up’: Constructing polyamorous languages in a culture of compulsory monogamy. Sexualities. 2006;9:584–601.

[75] Haupert M, Moors A, Gesselman A, Garcia J. Estimates and correlates of engagement in consensually non-monogamous relationships. Curr Sex Health Rep. 2017;9:155–165.

[76] Rubin JD, Moors AC, Mastick JL, Ziegler A, Conley TD. On the margins: Considering diversity among non-monogamous relationships. J fur Psychologie. 2014;22:1–23.

[77] Klesse C. (2014). Poly economics—capitalism, class, and polyamory. Inter J Politics, Culture, Soc. 2014;27(2):203–220.

[78] Weis D. L. The use of theory in sexuality research. J Sex Res. 1998;35(1):1–9.

