# Supplementary material for: A dyadic examination of self-determined sexual motives, need fulfillment, and relational outcomes among consensually non-monogamous partners
Source: PLoS One. 2021 Feb 16;16(2):e0247001. doi: 10.1371/journal.pone.0247001 (PMC7886188; doi:10.1371/journal.pone.0247001)
Supplement: S1 File — (PDF) [file pone.0247001.s001.pdf]

Online Supplement

Initial Background Survey

What year were you born?

1998
1997
1996
1995
1994
1993
1992
1991
1990
1989

Sometimes people identify themselves by race and/or ethnicity. Please check the group(s) with which you most identify (check all that apply).

American Indian or Alaska Native

Black, African American

White

Asian Indian

Chinese

Filipino

Japanese

Korean

Vietnamese

Native Hawaiian

Guamanian or Chomorro

Samoan

Mexican, Mexican American, Chicano

Puerto Rican

Cuban

Arab (e.g., Saudi Arabian, Iraqi)

West Asian (e.g., Iranian, Afgani)

Aboriginal/First Nations

[ ] Not listed, please specify:

Choose not to respond

My area of residence can be best described as:

Urban

Suburban

Rural

Neither

[ ] Other (specify)

Choose not to respond

What is the highest level of education that you have completed?

No schooling completed

Elementary school (grades 1-8)

Some high school

High school graduate

Some college/university

College/university graduate

Some trade/technical/vocational training

Trade/technical/vocational training degree or diploma

Some postgraduate work

Master's degree

Professional school degree (e.g., MD)

Doctoral degree

Choose not to respond

Do you have children?

Yes

No

Choose not to respond

If yes, how many children do you have?

If yes, do your children live with you at home?

Yes, full time

Yes, part time

Some children live at home, some do not

No

Choose not to respond

Which of these commonly used terms would you use to describe your sexual orientation?

Heterosexual

Gay

Lesbian

Bisexual

Queer

Pansexual

Uncertain or questioning

Asexual

My sexual orientation is not listed above. If your sexual orientation is not listed here, please tell us how you identify:

Choose not to respond

What sex were you assigned at birth?

Female

Male

Intersex

Choose not to respond

What is your current gender identity?

Woman (cisgender, transgender)

Man (cisgender, transgender)

Gender queer

Non-binary

Agender

My gender identity is not listed above

Choose not to respond

How did you hear about this survey?

Facebook

Twitter

Email listserv

Reddit

Other social media:

Other, please specify:

Well-Being

Below are five statements that you may agree or disagree with. Using the 1-7 scale below, indicate your agreement with each item by placing the appropriate number on the line preceding that item. Please be open and honest in your responding.

What is your agreement with the following items?

	**Strongly disagree 1**	**Disagree 2**	**Slightly disagree 3**	**Neither agree nor disagree 4**	**Slightly agree 5**	**Agree 6**	**Strongly agree 7**	Choose not to respond
In most ways my life is close to my ideal	○	○	○	○	○	○	○	○
The conditions of my life are excellent	○	○	○	○	○	○	○	○
I am satisfied with my life	○	○	○	○	○	○	○	○

	**Strongly disagree 1**	**Disagree 2**	**Slightly disagree 3**	**Neither agree nor disagree 4**	**Slightly agree 5**	**Agree 6**	**Strongly agree 7**	Choose not to respond
So far I have gotten the important things I want in life	○	○	○	○	○	○	○	○

	Strongly disagree 1	Disagree 2	Slightly disagree 3	Neither agree nor disagree 4	Slightly agree 5	Agree 6	Strongly agree 7	Choose not to respond
If I could live my life over, I would change almost nothing	○	○	○	○	○	○	○	○

Relational Questions

We will first ask you some questions about your relationship with your primary partner and once those are complete, we will ask you to report on your relationship with one additional partner if applicable.

My current relationship status is (check all that apply):

Casually dating one or more people

Open relationship (one or both of us has sex outside of the relationship consensually)

Polyamorous (one or both of us are in multiple loving and/or sexual relationships consensually)

Swinging relationship (one or both of you go to parties/clubs/etc., where partners may be exchanged for the night consensually)

Living with one partner, but not married or engaged

Living with multiple partners, but not married or engaged

Engaged to a partner

Engaged to more than one partner

Married to one partner

Married to more than one partner

[ ] Other: please specify

Choose not to respond

How long have you been in a romantic relationship with your current primary partner? (e.g., 7 years, 2 months)

What are the initials of your primary partner?

Other than your primary partner, how many additional relational and/or sexual partners do you currently have? **Please respond with whole numbers (e.g., 4, 5, etc.)** It is very important for us to have numerical data, **not** words like "four" or "five."

We know that many people have more than two partners, but for the purpose of this study, we would like you to report on up to one additional partner.

Please enter the initials of your additional partner - this will be inputted to the rest of the survey in order for you to identify which partner we are asking about.

Partner 2 Initials

Choose not to respond

What sex was your **primary partner's (initials: ${q://QID357/ChoiceTextEntryValue})** assigned at birth?

Female

Male

Intersex

Choose not to respond

What is your **primary partner's (initials: ${q://QID357/ChoiceTextEntryValue})** current gender identity?

Woman (cisgender, transgender)

Man (cisgender, transgender)

Gender queer

Non-binary

Agender

My gender identity is not listed above

Choose not to respond

How many times in the past month have you engaged in sexual activity with your **primary partner (initials: ${q://QID357/ChoiceTextEntryValue})**?

How long have you been in a romantic relationship with **partner 2 (initials: ${q://QID21/ChoiceTextEntryValue/2})**? (e.g., 7 years, 2 months)

What sex was **partner 2 (initials: ${q://QID21/ChoiceTextEntryValue/2})** assigned at birth?

Female

Male

Intersex

Choose not to respond

What is **partner 2's (initials: ${q://QID21/ChoiceTextEntryValue/2})** current gender identity?

Woman (cisgender, transgender)

Man (cisgender, transgender)

Gender queer

Non-binary

Agender

If your partner's gender identity is not listed above, please specify

Choose not to respond

How many times in the past month have you engaged in sexual activity with **partner 2 (initials: ${q://QID21/ChoiceTextEntryValue/2})**?

The last time you engaged in sexual activity with a partner, it was with...

My primary partner (initials: ${q://QID357/ChoiceTextEntryValue})

Partner 2 (initials: ${q://QID21/ChoiceTextEntryValue/2})

A partner that is not listed here

Choose not to respond

Is there anything else you would like to tell us about your relationship(s)?

Sexual Motives

People usually have many different reasons for engaging in sexual activity. Listed below are several statements that describe reasons you might have for engaging in sexual activity. For this task, we would like you to think about times you engaged in sexual activity with a partner(s). Use the scale below to indicate the extent to which each of the following statements reflects why you engaged in sexual activity with your partner(s).

In the past month, I engaged in sexual activity with my **primary partner (initials: ${q://QID357/ChoiceTextEntryValue})**...

	**Not at all for this reason 0**	**A little for this reason 1**	**Somewhat for this reason 2**	**Quite a bit for this reason 3**	**Very much for this reason 4**	Choose not to respond
Because I expected it to be interesting and exciting.	○	○	○	○	○	○
For the pleasure of sharing a special and intimate experience.	○	○	○	○	○	○
Because I value sex as part of a full life.	○	○	○	○	○	○
Because my sex drive was high, and I felt like I needed to have sex.	○	○	○	○	○	○
Because I felt pressured.	○	○	○	○	○	○

	**Not at all for this reason 0**	**A little for this reason 1**	**Somewhat for this reason 2**	**Quite a bit for this reason 3**	**Very much for this reason 4**	Choose not to respond
Because I wanted sex to be a celebration of the feelings I share with my partner.	○	○	○	○	○	○
Because I expect the pleasure of my physical satisfaction.	○	○	○	○	○	○

	Not at all for this reason 0	A little for this reason 1	Somewhat for this reason 2	Quite a bit for this reason 3	Very much for this reason 4	Choose not to respond
Because I thought my partner (s) would like me better, or be happier with me.	O	O	O	O	O	O
Because I wanted to feel more powerful or dominant.	O	O	O	O	O	O
Because it feels good.	O	O	O	O	O	O

In the past month, I engaged in sexual activity with my **primary partner (initials: ${q://QID357/ChoiceTextEntryValue})**...

	Not at all for this reason 0	A little for this reason 1	Somewhat for this reason 2	Quite a bit for this reason 3	Very much for this reason 4	Choose not to respond
Because it is exciting to be sexually intimate with my partner(s)	O	O	O	O	O	O
But I did not feel like I was in control of my own behavior	O	O	O	O	O	O
Because it is stimulating and enjoyable.	O	O	O	O	O	O
Because my body ached to have sex.	O	O	O	O	O	O
Because I would feel anxious or guilty if I didn't go along.	O	O	O	O	O	O

	Not at all for this reason 0	A little for this reason 1	Somewhat for this reason 2	Quite a bit for this reason 3	Very much for this reason 4	Choose not to respond
Because it helped me relax or get to sleep.	O	O	O	O	O	O

	Not at all for this reason 0	A little for this reason 1	Somewhat for this reason 2	Quite a bit for this reason 3	Very much for this reason 4	Choose not to respond
Because sex is an important part of my relationship(s)	O	O	O	O	O	O
Because I didn't want to say no.	O	O	O	O	O	O
Because I expect a satisfyingly deep connection with my partner(s) during sex.	O	O	O	O	O	O
Because I need to relieve myself of the tension and stress of the day.	O	O	O	O	O	O

In the past month, I engaged in sexual activity with my **primary partner (initials: ${q://QID357/ChoiceTextEntryValue})**...

	Not at all for this reason 0	A little for this reason 1	Somewhat for this reason 2	Quite a bit for this reason 3	Very much for this reason 4	Choose not to respond
Because I see sex as a healthy activity.	O	O	O	O	O	O
Because I wanted to enjoy the physical sensations.	O	O	O	O	O	O
Because I wanted to show that I am capable of performing.	O	O	O	O	O	O
Because I enjoy knowing my partner(s) this way.	O	O	O	O	O	O
Because I see sex as an important part of who I am.	O	O	O	O	O	O

	Not at all for this reason 0	A little for this reason 1	Somewhat for this reason 2	Quite a bit for this reason 3	Very much for this reason 4	Choose not to respond
	Not at all for this reason 0	A little for this reason 1	Somewhat for this reason 2	Quite a bit for this reason 3	Very much for this reason 4	Choose not to respond
Because I worry I will be punished or neglected if I don't.	O	O	O	O	O	O
Because I felt driven to have sex.	O	O	O	O	O	O
Because the proposition made me feel more attractive.	O	O	O	O	O	O
Because I wanted a fun experience.	O	O	O	O	O	O
Because I thought sex would get my something I wanted later.	O	O	O	O	O	O

In the past month, I engaged in sexual activity with my **primary partner (initials: ${q://QID357/ChoiceTextEntryValue})**...

	Not at all for this reason 0	A little for this reason 1	Somewhat for this reason 2	Quite a bit for this reason 3	Very much for this reason 4	Choose not to respond
But I don't know why. It just happened.	O	O	O	O	O	O
Because I wanted to share a mutually pleasurable activity.	O	O	O	O	O	O
Because I think it is a healthy aspect of my relationship(s).	O	O	O	O	O	O
Because I want another person to be under my control.	O	O	O	O	O	O

	Not at all for this reason 0	A little for this reason 1	Somewhat for this reason 2	Quite a bit for this reason 3	Very much for this reason 4	Choose not to respond
Because I think sex is an enjoyable way to share our feelings.	O	O	O	O	O	O

	Not at all for this reason 0	A little for this reason 1	Somewhat for this reason 2	Quite a bit for this reason 3	Very much for this reason 4	Choose not to respond
Because I worried my partner(s) might reject me if I didn't.	O	O	O	O	O	O
Because I would feel bad to withhold sex.	O	O	O	O	O	O
Because I wanted to enjoy being close to my friend.	O	O	O	O	O	O
Because I needed to orgasm.	O	O	O	O	O	O
Because I enjoy being sexual.	O	O	O	O	O	O

In the past month, I engaged in sexual activity with my **primary partner (initials: ${q://QID357/ChoiceTextEntryValue})**...

	Not at all for this reason 0	A little for this reason 1	Somewhat for this reason 2	Quite a bit for this reason 3	Very much for this reason 4	Choose not to respond
Because I value sex as an important part of maintaining a good friendship.	O	O	O	O	O	O
Because I thought my partner(s) would treat me better afterward.	O	O	O	O	O	O

	Not at all for this reason 0	A little for this reason 1	Somewhat for this reason 2	Quite a bit for this reason 3	Very much for this reason 4	Choose not to respond
Because I want to enjoy the closeness of being physically joined with my sexual partner(s).	○	○	○	○	○	○
Because sex makes me feel better about myself.	○	○	○	○	○	○

	Not at all for this reason 0	A little for this reason 1	Somewhat for this reason 2	Quite a bit for this reason 3	Very much for this reason 4	Choose not to respond
Because I wanted to show how good I am in bed.	○	○	○	○	○	○
Because I think saying no will start a conflict.	○	○	○	○	○	○
Because alcohol makes me lose control.	○	○	○	○	○	○
Because I thought sex will make me feel more secure.	○	○	○	○	○	○

	Not at all for this reason 0	A little for this reason 1	Somewhat for this reason 2	Quite a bit for this reason 3	Very much for this reason 4	Choose not to respond
Because I value how sex can bring me closer to another person(s).	○	○	○	○	○	○
Because my sexual desire was high.	○	○	○	○	○	○
Because I needed to relieve myself of sexual tension.	○	○	○	○	○	○
But I have no idea why I did.	○	○	○	○	○	○

Is there anything else you would like us to know about the reasons you engage in sexual activity with a partner(s)?

Sexual Need Satisfaction

Now we will ask you about how you feel when you have sex with your **primary relationship (initials: ${e://Field/PrimaryInitials}).**

Please respond to each statement by indicating how true it is for you.

When I have sex with my **primary relationship (initials: ${e://Field/PrimaryInitials})...**

	**Not at all true 1**	**2**	**3**	**Somewhat True 4**	**5**	**6**	**Very True 7**	Choose not to respond
I feel free to be who I am.	○	○	○	○	○	○	○	○
I feel like a competent person.	○	○	○	○	○	○	○	○
I feel loved and cared about.	○	○	○	○	○	○	○	○
I often feel inadequate or incompetent.	○	○	○	○	○	○	○	○
I have a say in what happens, and I can voice my opinion.	○	○	○	○	○	○	○	○

	Not at all true 1	2	3	Somewhat True 4	5	6	Very True 7	Choose not to respond
	Not at all true 1	2	3	Somewhat True 4	5	6	Very True 7	Choose not to respond
I often feel a lot of distance in our relationship.	○	○	○	○	○	○	○	○
I feel very capable and effective.	○	○	○	○	○	○	○	○
I feel a lot of closeness and intimacy.	○	○	○	○	○	○	○	○
I feel controlled and pressured to be certain ways.	○	○	○	○	○	○	○	○

Relationship Satisfaction: Dyadic Adjustment Scale - 4

Please answer the following questions while thinking of your **primary relationship (initials: ${e://Field/PrimaryInitials}**), using the following scale.

	Never 0	1	2	3	4	All the time 5	Choose not to respond
How often do you discuss or have you considered divorce, separation, or terminating your relationship?	○	○	○	○	○	○	○
In general, how often do you think that things between you and your partner are going well?	○	○	○	○	○	○	○
Do you confide in your partner?	○	○	○	○	○	○	○

The following represents different degrees of happiness in your relationship. Please select the choice which best describes the degree of happiness, all things considered, of your relationship.

0 Extremely unhappy

1 Fairly unhappy

2 A little unhappy

3 Happy

4 Very happy

5 Extremely happy

6 Perfect

Choose not to respond

Relationship Satisfaction Primary

Please complete the following questions about your **primary relationship (initials: ${e://Field/PrimaryInitials}**).

	**Very strong disagreement 1**	**2**	**3**	**4**	**5**	**6**	**Very strong agreement 7**	Choose not to respond
Right now my relationship with my primary partner is stable.								
Right now my primary relationship is strong.								
Right now my relationship with my primary partner makes me happy.								

	**Very strong disagreement 1**	**2**	**3**	**4**	**5**	**6**	**Very strong agreement 7**	Choose not to respond

	Very strong disagreement 1	2	3	4	5	6	Very strong agreement 7	Choose not to respond
Right now I am experiencing conflict with my primary partner.	O	O	O	O	O	O	O	O
Right now I am unsure if my relationship with my primary partner will last.	O	O	O	O	O	O	O	O
Right now my primary partner and I are getting on each other's nerves.	O	O	O	O	O	O	O	O

Overall Sexual Satisfaction Primary

Overall, how satisfied are you with the sexual aspect of your **primary relationship (initials: ${e://Field/PrimaryInitials}**) relationship?

Not at all satisfied

A little satisfied

Moderately satisfied

Very satisfied

Extremely satisfied

Choose not to respond

Is there anything else you would like to tell us about sexual satisfaction within this relationship?

Sexual Satisfaction (NSSS-S)

Thinking about your sex life with your **primary relationship (initials: ${e://Field/PrimaryInitials}**) during the last six months, please rate your satisfaction with the following aspects:

	**Not at all satisfied 1**	**A little satisfied 2**	**Moderately satisfied 3**	**Very satisfied 4**	**Extremely satisfied 5**	Choose not to respond
The quality of my orgasms	O	O	O	O	O	O
My "letting go" and surrender to sexual pleasure during sex	O	O	O	O	O	O
The way I sexually react to my partner	O	O	O	O	O	O
My body's sexual functioning	O	O	O	O	O	O
	**Not at all satisfied 1**	**A little satisfied 2**	**Moderately satisfied 3**	**Very satisfied 4**	**Extremely satisfied 5**	Choose not to respond
My mood after sexual activity	O	O	O	O	O	O
The pleasure I provide to my partner	O	O	O	O	O	O
The balance between what I give and receive in sex	O	O	O	O	O	O
My partner's emotional opening up during sex	O	O	O	O	O	O

	**Not at all satisfied 1**	**A little satisfied 2**	**Moderately satisfied 3**	**Very satisfied 4**	**Extremely satisfied 5**	Choose not to respond
	**Not at all satisfied 1**	**A little satisfied 2**	**Moderately satisfied 3**	**Very satisfied 4**	**Extremely satisfied 5**	Choose not to respond
My partner's ability to orgasm	○	○	○	○	○	○
My partner's sexual creativity	○	○	○	○	○	○
The variety of my sexual activities	○	○	○	○	○	○
The frequency of my sexual activity	○	○	○	○	○	○

Sexual Communal Strength Primary Partner

Please rate the following items about your **primary relationship (initials: ${e://Field/PrimaryInitials}**)

	**Not at all 0**	**1**	**2**	**3**	**Extremely 4**	Choose not to respond
How far would you be willing to go to meet your primary partner's (initials: ${q://QID357/ChoiceTextEntryValue}) sexual needs?	○	○	○	○	○	○
How readily can you put the sexual needs of your primary partner's (initials: ${q://QID357/ChoiceTextEntryValue}) out of your thoughts?	○	○	○	○	○	○
How high a priority for you is meeting the sexual needs of your primary partner (initials: ${q://QID357/ChoiceTextEntryValue})?	○	○	○	○	○	○
How easily could you accept not meeting your primary partner's (initials: ${q://QID357/ChoiceTextEntryValue}) sexual needs?	○	○	○	○	○	○

	**Not at all 0**	**1**	**2**	**3**	**Extremely 4**	Choose not to respond
	**Not at all 0**	**1**	**2**	**3**	**Extremely 4**	Choose not to respond
How likely are you to sacrifice your own needs to meet the sexual needs of your primary partner (initials: ${q://QID357/ChoiceTextEntryValue})?	○	○	○	○	○	○
Please select response option 4 (Extremely)	○	○	○	○	○	○
How happy do you feel when satisfying your primary partner's (initials: ${q://QID357/ChoiceTextEntryValue}) sexual needs?	○	○	○	○	○	○

Self-Other Primary Partner

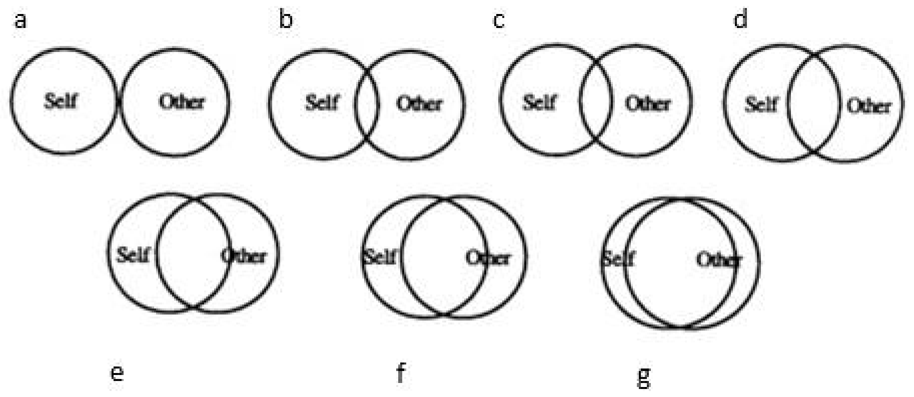

Please chose which picture best describes your relationship with your ***primary relationship (initials: ${e://Field/PrimaryInitials}*** *)*

a

b

c

d

e

f

g

Choose not to respond

Dyadic Sexual Communication Scale (short)

Thinking about your **primary relationship (initials: ${e://Field/PrimaryInitials}),** please indicate how strongly you disagree or agree with each of these items.

	**Disagree strongly 1**	**2**	**3**	**4**	**5**	**Agree strongly 6**	Choose not to respond
I find some sexual matters are too upsetting to talk about with my primary partner.	○	○	○	○	○	○	○
I think it is difficult for my primary partner to tell me what he/she/they like(s) to do sexually.	○	○	○	○	○	○	○
It is easy for me to tell my primary partner what to do or don't like to do during sex.	○	○	○	○	○	○	○
	**Disagree strongly 1**	**2**	**3**	**4**	**5**	**Agree strongly 6**	Choose not to respond
My primary partner hardly ever talks to me when I want to talk about our sex life.	○	○	○	○	○	○	○
My primary partner really cares about what I think about sex.	○	○	○	○	○	○	○
Talking about sex with my primary partner is usually fun for both of us.	○	○	○	○	○	○	○

Additional Communication Questions

Please think about your communication with your **primary relationship (initials: ${e://Field/PrimaryInitials}**)

I find it easy to talk to my primary partner about problems or concerns within our relationship

1 Strongly disagree

2

3

4

5 Strongly agree

Choose not to respond

To what degree have you and your primary partner discussed the boundaries or "rules" related to sexual and/or emotional connections with other people?

A great deal

Somewhat

Not very much

Not at all

Choose not to respond

Compared to most people you know, do you feel the QUALITY of the communication between you and your primary partner is generally…

1 Well below average

2 Below average

3 About average

4 Above average

5 Well above average

Choose not to respond

Is there anything else you would like us to know about communication with your relationship?

Overall Need Fulfillment

People look for different qualities in romantic relationships. Think about your current romantic relationship with your **primary relationship (initials: ${e://Field/PrimaryInitials}**). below is a list of relationship qualities.

Please use the following scale to indicate how true each quality is in your current relationship with your **primary relationship (initials: ${e://Field/PrimaryInitials}**).

	**Never true 1**	**2**	**3**	**4**	**5**	**6**	**Always True 7**	Choose not to respond
I feel closeness and intimacy with my primary partner (initials: ${e://Field/PrimaryInitials})	○	○	○	○	○	○	○	○
I can honestly express my thoughts and feelings to my primary partner (initials: ${e://Field/PrimaryInitials})	○	○	○	○	○	○	○	○
My primary partner (initials: ${e://Field/PrimaryInitials}) really understands me	○	○	○	○	○	○	○	○

	**Never true 1**	**2**	**3**	**4**	**5**	**6**	**Always True 7**	Choose not to respond
	**Never true 1**	**2**	**3**	**4**	**5**	**6**	**Always True 7**	Choose not to respond
My primary partner (initials: ${e://Field/PrimaryInitials}) and I enjoy spending time together	○	○	○	○	○	○	○	○
My primary partner (initials: ${e://Field/PrimaryInitials}) and I laugh and have a good time together	○	○	○	○	○	○	○	○
My primary partner (initials: ${e://Field/PrimaryInitials}) and I share our beliefs and values	○	○	○	○	○	○	○	○

Please use the following scale to indicate how true each quality is in your current relationship with your **primary relationship (initials: ${e://Field/PrimaryInitials}**).

	**Never true 1**	**2**	**3**	**4**	**5**	**6**	**Always True 7**	Choose not to respond
I feel good about myself with my primary partner (initials: ${e://Field/PrimaryInitials})	○	○	○	○	○	○	○	○
My primary partner (initials: ${e://Field/PrimaryInitials}) brings out the best in me	○	○	○	○	○	○	○	○
	**Never true 1**	**2**	**3**	**4**	**5**	**6**	**Always True 7**	Choose not to respond

	**Never true 1**	**2**	**3**	**4**	**5**	**6**	**Always True 7**	Choose not to respond
My primary partner (initials: ${e://Field/PrimaryInitials}) makes me feel cherished and special	○	○	○	○	○	○	○	○
I like the way my primary partner (initials: ${e://Field/PrimaryInitials}) makes me feel about myself	○	○	○	○	○	○	○	○

Please use the following scale to indicate how true each quality is in your current relationship with your **primary relationship (initials: ${e://Field/PrimaryInitials}**).

	**Never true 1**	**2**	**3**	**4**	**5**	**6**	**Always True 7**	Choose not to respond
I feel free to be who I am with my primary partner (initials: ${e://Field/PrimaryInitials})	○	○	○	○	○	○	○	○
My primary partner (initials: ${e://Field/PrimaryInitials}) respects my choices and decisions	○	○	○	○	○	○	○	○
My primary partner (initials: ${e://Field/PrimaryInitials}) respects me	○	○	○	○	○	○	○	○

	**Never true 1**	**2**	**3**	**4**	**5**	**6**	**Always True 7**	Choose not to respond
My primary partner (initials: ${e://Field/PrimaryInitials}) supports that I do things on my own	○	○	○	○	○	○	○	○
My primary partner (initials: ${e://Field/PrimaryInitials}) respects my independence	○	○	○	○	○	○	○	○

Please use the following scale to indicate how true each quality is in your current relationship with your **primary relationship (initials: ${e://Field/PrimaryInitials}**).

	Never true 1	2	3	4	5	6	Always True 7	Choose not to respond
I have exciting experiences with my primary partner (initials: ${e://Field/PrimaryInitials})	◯	◯	◯	◯	◯	◯	◯	◯
I have a variety of new experiences with my primary partner (initials: ${e://Field/PrimaryInitials})	◯	◯	◯	◯	◯	◯	◯	◯
I learn new things from my primary partner (initials: ${e://Field/PrimaryInitials})	◯	◯	◯	◯	◯	◯	◯	◯
	**Never true 1**	**2**	**3**	**4**	**5**	**6**	**Always True 7**	Choose not to respond
I am spontaneous with my primary partner (initials: ${e://Field/PrimaryInitials})	◯	◯	◯	◯	◯	◯	◯	◯
My relationship with my primary partner (initials: ${e://Field/PrimaryInitials}) allows me to see the world in new ways	◯	◯	◯	◯	◯	◯	◯	◯

Please use the following scale to indicate how true each quality is in your current relationship with your **primary relationship (initials: ${e://Field/PrimaryInitials}**).

	Never true 1	2	3	4	5	6	Always True 7	Choose not to respond
My sex life with my primary partner (initials: ${e://Field/PrimaryInitials}) is exciting	◯	◯	◯	◯	◯	◯	◯	◯

	Never true 1	2	3	4	5	6	Always True 7	Choose not to respond
I engage in desired sexual activities with my primary partner (initials: ${e://Field/PrimaryInitials})	○	○	○	○	○	○	○	○
My primary partner (initials: ${e://Field/PrimaryInitials}) and I try new things sexually	○	○	○	○	○	○	○	○

	Never true 1	2	3	4	5	6	Always True 7	Choose not to respond
I share my sexual fantasies with my primary partner (initials ${e://Field/PrimaryInitials})	○	○	○	○	○	○	○	○
My primary partner (initials: ${e://Field/PrimaryInitials}) understands my sexual needs	○	○	○	○	○	○	○	○

Please use the following scale to indicate how true each quality is in your current relationship with your **primary relationship (initials: ${e://Field/PrimaryInitials}**).

	Never true 1	2	3	4	5	6	Always True 7	Choose not to respond
I feel secure in my relationship with my primary partner (initials: ${e://Field/PrimaryInitials})	○	○	○	○	○	○	○	○
My relationship with my primary partner (initials: ${e://Field/PrimaryInitials}) is stable	○	○	○	○	○	○	○	○
I can count on my primary partner (initials: ${e://Field/PrimaryInitials})	○	○	○	○	○	○	○	○

	**Never true 1**	**2**	**3**	**4**	**5**	**6**	**Always True 7**	Choose not to respond
	**Never true 1**	**2**	**3**	**4**	**5**	**6**	**Always True 7**	Choose not to respond
My primary partner (initials: ${e://Field/PrimaryInitials}) will always be there for me	○	○	○	○	○	○	○	○
I trust my primary partner (initials: ${e://Field/PrimaryInitials})	○	○	○	○	○	○	○	○

Please use the following scale to indicate how true each quality is in your current relationship with your **primary relationship (initials: ${e://Field/PrimaryInitials}**).

	**Never true 1**	**2**	**3**	**4**	**5**	**6**	**Always True 7**	Choose not to respond
My primary partner (initials: ${e://Field/PrimaryInitials}) is there for me when I need comfort	○	○	○	○	○	○	○	○
I can talk to my primary partner (initials: ${e://Field/PrimaryInitials}) when I'm worried about something	○	○	○	○	○	○	○	○
My primary partner (initials: ${e://Field/PrimaryInitials}) is understanding when I'm upset	○	○	○	○	○	○	○	○
My primary partner (initials: ${e://Field/PrimaryInitials}) supports me emotionally	○	○	○	○	○	○	○	○
My primary partner (initials: ${e://Field/PrimaryInitials}) comforts and supports me when I am stressed	○	○	○	○	○	○	○	○

Is there anything else you would like to tell us about need fulfillment within your relationship?

Relationship Goals

In my relationship with my primary partner (initials: ${e://Field/PrimaryInitials})...

	Strongly disagree 1	2	3	4	5	6	Strongly agree 7	Choose not to respond
I am trying to avoid getting embarrassed, betrayed, or hurt by my romantic partner	○	○	○	○	○	○	○	○
I am trying to enhance the bonding and intimacy in my romantic relationship	○	○	○	○	○	○	○	○
I am trying to avoid disagreements and conflicts with my romantic partner	○	○	○	○	○	○	○	○
I am trying to deepen my relationships with my romantic partner	○	○	○	○	○	○	○	○
	Strongly disagree 1	2	3	4	5	6	Strongly agree 7	Choose not to respond

	Strongly disagree 1	2	3	4	5	6	Strongly agree 7	Choose not to respond
I am trying to make sure that nothing bad happens in my romantic relationship	◯	◯	◯	◯	◯	◯	◯	◯
I am trying to share many fun and meaningful experiences with my romantic partner	◯	◯	◯	◯	◯	◯	◯	◯
I am trying to stay away from situations that could harm my romantic relationship	◯	◯	◯	◯	◯	◯	◯	◯
I am trying to move toward growth and development in my romantic relationship	◯	◯	◯	◯	◯	◯	◯	◯

Compersion Questions

Sometimes people in the polyamorous community report feeling something that is the opposite of jealousy. Compersion is described as having positive feelings when a partner experiences pleasure with other people. How often do you experience compersion within your relationship(s)?

Never

A few times a year

Once a month

Two or three times a month

Once a week

Two or three times a week

Four or more times a week

Choose not to respond

Now we are going to ask about a number of things that can occur in relationships. Listed below are a number of emotions that typically represent how people may feel. If

you have experienced any of these, answer with those experiences in mind. If you have never experienced the specific thing asked about, imagine instead what you think it would feel like. For each item, use the scale to signify how much of each of the emotions you felt or think you would feel.

If my **primary partner (initials: ${e://Field/PrimaryInitials})** fell in love with another person, I would feel…

	**Not at all** 1	2	3	**Neutral** 4	5	6	**Very much** 7	Choose not to respond
Happy	○	○	○	○	○	○	○	○
Sad	○	○	○	○	○	○	○	○
Love for my partner	○	○	○	○	○	○	○	○
Angry	○	○	○	○	○	○	○	○
Enthusiastic	○	○	○	○	○	○	○	○

	**Not at all** 1	2	3	**Neutral** 4	5	6	**Very much** 7	Choose not to respond
Jealous	○	○	○	○	○	○	○	○
Joyful	○	○	○	○	○	○	○	○
Loved by my partner	○	○	○	○	○	○	○	○
Scared	○	○	○	○	○	○	○	○
Excited for my partner	○	○	○	○	○	○	○	○

Has this ever happened to you with your **primary partner (initials: ${e://Field/PrimaryInitials})**?

Yes

No

Choose not to respond

Other

If I watched my **primary partner (initials: ${e://Field/PrimaryInitials})** expressing intense sexual desire for another person, I would feel…

	**Not at all 1**	**2**	**3**	**Neutral 4**	**5**	**6**	**Very much 7**	Choose not to respond
Happy	○	○	○	○	○	○	○	○
Sad	○	○	○	○	○	○	○	○
Love for my partner	○	○	○	○	○	○	○	○
Angry	○	○	○	○	○	○	○	○
Enthusiastic	○	○	○	○	○	○	○	○

	**Not at all 1**	**2**	**3**	**Neutral 4**	**5**	**6**	**Very much 7**	Choose not to respond
Jealous	○	○	○	○	○	○	○	○
Joyful	○	○	○	○	○	○	○	○
Loved by my partner	○	○	○	○	○	○	○	○
Scared	○	○	○	○	○	○	○	○
Excited for my partner	○	○	○	○	○	○	○	○

Has this ever happened to you with your **primary partner (initials: ${e://Field/PrimaryInitials})**?

Yes

No

Choose not to respond

Other

Sexual Motives P2

Now we will ask many of the same questions about **partner 2 (initials: ${e://Field/SecondaryInitials}).**

People usually have many different reasons for engaging in sexual activity. Listed below are several statements that describe reasons you might have for engaging in sexual activity. For this task, we would like you to think about times you engaged in sexual activity with a partner(s). Use the scale below to indicate the extent to which each of the following statements reflects why you engaged in sexual activity with your partner(s).

In the past month, I engaged in sexual activity with **partner 2 (initials: ${e://Field/SecondaryInitials})...**

	**Not at all for this reason 0**	**A little for this reason 1**	**Somewhat for this reason 2**	**Quite a bit for this reason 3**	**Very much for this reason 4**	Choose not to respond
Because I expected it to be interesting and exciting.	O	O	O	O	O	O
For the pleasure of sharing a special and intimate experience.	O	O	O	O	O	O

	Not at all for this reason 0	A little for this reason 1	Somewhat for this reason 2	Quite a bit for this reason 3	Very much for this reason 4	Choose not to respond
Because I value sex as part of a full life.	O	O	O	O	O	O
Because my sex drive was high, and I felt like I needed to have sex.	O	O	O	O	O	O
Because I felt pressured.	O	O	O	O	O	O

	Not at all for this reason 0	A little for this reason 1	Somewhat for this reason 2	Quite a bit for this reason 3	Very much for this reason 4	Choose not to respond
Because I wanted sex to be a celebration of the feelings I share with my partner.	O	O	O	O	O	O
Because I expect the pleasure of my physical satisfaction.	O	O	O	O	O	O
Because I thought my partner (s) would like me better, or be happier with me.	O	O	O	O	O	O
Because I wanted to feel more powerful or dominant.	O	O	O	O	O	O
Because it feels good.	O	O	O	O	O	O

In the past month, I engaged in sexual activity with **partner 2 (initials: ${e://Field/SecondaryInitials})...**

	Not at all for this reason 0	A little for this reason 1	Somewhat for this reason 2	Quite a bit for this reason 3	Very much for this reason 4	Choose not to respond

Because it is exciting to be sexually intimate with my partner(s).	O	O	O	O	O	O
But I did not feel like I was in control of my own behavior.	O	O	O	O	O	O
Because it is stimulating and enjoyable.	O	O	O	O	O	O
Because my body ached to have sex.	O	O	O	O	O	O
Because I would feel anxious or guilty if I didn't go along.	O	O	O	O	O	O
	**Not at all for this reason 0**	**A little for this reason 1**	**Somewhat for this reason 2**	**Quite a bit for this reason 3**	**Very much for this reason 4**	Choose not to respond
Because it helped me relax or get to sleep.	O	O	O	O	O	O
Because sex is an important part of my relationship(s).	O	O	O	O	O	O
Because I didn't want to say no.	O	O	O	O	O	O
Because I expect a satisfyingly deep connection with my partner(s) during sex.	O	O	O	O	O	O
Because I need to relieve myself of the tension and stress of the day.	O	O	O	O	O	O

In the past month, I engaged in sexual activity with **partner 2 (initials: ${e://Field/SecondaryInitials})...**

	Not at all for this reason 0	A little for this reason 1	Somewhat for this reason 2	Quite a bit for this reason 3	Very much for this reason 4	Choose not to respond

Because I see sex as a healthy activity.	O	O	O	O	O	O
Because I wanted to enjoy the physical sensations.	O	O	O	O	O	O
Because I wanted to show that I am capable of performing.	O	O	O	O	O	O
Because I enjoy knowing my partner(s) this way.	O	O	O	O	O	O
Because I see sex as an important part of who I am.	O	O	O	O	O	O
	**Not at all for this reason 0**	**A little for this reason 1**	**Somewhat for this reason 2**	**Quite a bit for this reason 3**	**Very much for this reason 4**	Choose not to respond
Because I worry I will be punished or neglected if I don't.	O	O	O	O	O	O
Because I felt driven to have sex.	O	O	O	O	O	O
Because the proposition made me feel more attractive.	O	O	O	O	O	O
Because I wanted a fun experience.	O	O	O	O	O	O
Because I thought sex would get my something I wanted later.	O	O	O	O	O	O

In the past month, I engaged in sexual activity with **partner 2 (initials: ${e://Field/SecondaryInitials})...**

	Not at all for this reason 0	A little for this reason 1	Somewhat for this reason 2	Quite a bit for this reason 3	Very much for this reason 4	Choose not to respond

But I don't know why. It just happened.	O	O	O	O	O	O
Because I wanted to share a mutually pleasurable activity.	O	O	O	O	O	O
Because I think it is a healthy aspect of my relationship(s).	O	O	O	O	O	O
Because I want another person to be under my control.	O	O	O	O	O	O
Because I think sex is an enjoyable way to share our feelings.	O	O	O	O	O	O
	**Not at all for this reason 0**	**A little for this reason 1**	**Somewhat for this reason 2**	**Quite a bit for this reason 3**	**Very much for this reason 4**	Choose not to respond
Because I worried my partner(s) might reject me if I didn't.	O	O	O	O	O	O
Because I would feel bad to withhold sex.	O	O	O	O	O	O
Because I wanted to enjoy being close to my friend.	O	O	O	O	O	O
Because I needed to orgasm.	O	O	O	O	O	O
Because I enjoy being sexual.	O	O	O	O	O	O

In the past month, I engaged in sexual activity with **partner 2 (initials: ${e://Field/SecondaryInitials})...**

	Not at all for this reason 0	A little for this reason 1	Somewhat for this reason 2	Quite a bit for this reason 3	Very much for this reason 4	Choose not to respond
Because I value sex as an important part of maintaining a good friendship.	O	O	O	O	O	O

	Not at all for this reason 0	A little for this reason 1	Somewhat for this reason 2	Quite a bit for this reason 3	Very much for this reason 4	Choose not to respond
Because I thought my partner(s) would treat me better afterward.	○	○	○	○	○	○
Because I want to enjoy the closeness of being physically joined with my sexual partner(s).	○	○	○	○	○	○
Because sex makes me feel better about myself.	○	○	○	○	○	○

	Not at all for this reason 0	A little for this reason 1	Somewhat for this reason 2	Quite a bit for this reason 3	Very much for this reason 4	Choose not to respond
Because I wanted to show how good I am in bed.	○	○	○	○	○	○
Because I think saying no will start a conflict.	○	○	○	○	○	○
Because alcohol makes me lose control.	○	○	○	○	○	○
Because I thought sex will make me feel more secure.	○	○	○	○	○	○

	Not at all for this reason 0	A little for this reason 1	Somewhat for this reason 2	Quite a bit for this reason 3	Very much for this reason 4	Choose not to respond
Because I value how sex can bring me closer to another person(s).	○	○	○	○	○	○
Because my sexual desire was high.	○	○	○	○	○	○
Because I needed to relieve myself of sexual tension.	○	○	○	○	○	○

	Not at all for this reason 0	A little for this reason 1	Somewhat for this reason 2	Quite a bit for this reason 3	Very much for this reason 4	Choose not to respond
But I have no idea why I did.	O	O	O	O	O	O

Is there anything else you would like us to know about the reasons you engage in sexual activity with a partner(s)?

Sexual Need Satisfaction P2

Please respond to each statement by indicating how true it is for you.

When I have sex with **partner 2 (initials: ${e://Field/SecondaryInitials})...**

	Not at all true 1	2	3	Somewhat True 4	5	6	Very True 7	Choose not to respond
I feel free to be who I am.	O	O	O	O	O	O	O	O
I feel like a competent person.	O	O	O	O	O	O	O	O
I feel loved and cared about.	O	O	O	O	O	O	O	O
I often feel inadequate or incompetent.	O	O	O	O	O	O	O	O

	Not at all true 1	2	3	Somewhat True 4	5	6	Very True 7	Choose not to respond
I have a say in what happens, and I can voice my opinion.	O	O	O	O	O	O	O	O

	Not at all true 1	2	3	Somewhat True 4	5	6	Very True 7	Choose not to respond
I often feel a lot of distance in our relationship.	O	O	O	O	O	O	O	O
I feel very capable and effective.	O	O	O	O	O	O	O	O
I feel a lot of closeness and intimacy.	O	O	O	O	O	O	O	O
I feel controlled and pressured to be certain ways.	O	O	O	O	O	O	O	O

Relationship Satisfaction Partner 2

Please rate how strongly you agree or disagree with the following items while thinking about **partner 2 (initials: ${e://Field/SecondaryInitials})**.

	Very strong disagreement 1	2	3	4	5	6	Very strong agreement 7	Choose not to respond
Right now my relationship with partner 2 (initials: ${e://Field/SecondaryInitials}) is stable.	O	O	O	O	O	O	O	O
Right now my relationship with partner 2 (initials: ${e://Field/SecondaryInitials}) is strong.	O	O	O	O	O	O	O	O
Right now my relationship withpartner 2 (initials: ${e://Field/SecondaryInitials}) makes me happy.	O	O	O	O	O	O	O	O

	Very strong disagreement 1	2	3	4	5	6	Very strong agreement 7	Choose not to respond

	Very strong disagreement 1	2	3	4	5	6	Very strong agreement 7	Choose not to respond
Right now I am experiencing conflict with partner 2 (initials: ${e://Field/SecondaryInitials}).	○	○	○	○	○	○	○	○
Right now I am unsure if my relationship with partner 2 (initials: ${e://Field/SecondaryInitials}) will last.	○	○	○	○	○	○	○	○
Right now partner 2 (initials: ${e://Field/SecondaryInitials}) and I are getting on each other's nerves.	○	○	○	○	○	○	○	○

Sexual Communal Strength Partner 2

Please rate the following items about **partner 2 (initials: ${e://Field/SecondaryInitials})**

	Not at all 0	1	2	3	Extremely 4	Choose not to respond
How far would you be willing to go to meet partner 2 (initials: ${e://Field/SecondaryInitials}) sexual needs?	○	○	○	○	○	○
How readily can you put the sexual needs of partner 2 (initials: ${e://Field/SecondaryInitials}) out of your thoughts?	○	○	○	○	○	○
How high a priority for you is meeting the sexual needs of partner 2 (initials: ${e://Field/SecondaryInitials})?	○	○	○	○	○	○

	Not at all 0	1	2	3	Extremely 4	Choose not to respond
	**Not at all 0**	**1**	**2**	**3**	**Extremely 4**	Choose not to respond
How easily could you accept not meeting partner 2 (initials: ${e://Field/SecondaryInitials}) sexual needs?	○	○	○	○	○	○
How likely are you to sacrifice your own needs to meet the sexual needs of partner 2 (initials: ${e://Field/SecondaryInitials})?	○	○	○	○	○	○
How happy do you feel when satisfying partner 2 (initials: ${e://Field/SecondaryInitials}) sexual needs?	○	○	○	○	○	○

Overall Sexual Satisfaction Partner 2

Overall how satisfied are you with the sexual aspect of your relationship with **partner 2 (initials: ${e://Field/SecondaryInitials})?**

Not at all satisfied

A little satisfied

Moderately satisfied

Very satisfied

Extremely satisfied

Choose not to respond

Is there anything else you would like to tell us about sexual satisfaction within this relationship?

Self-Other P2

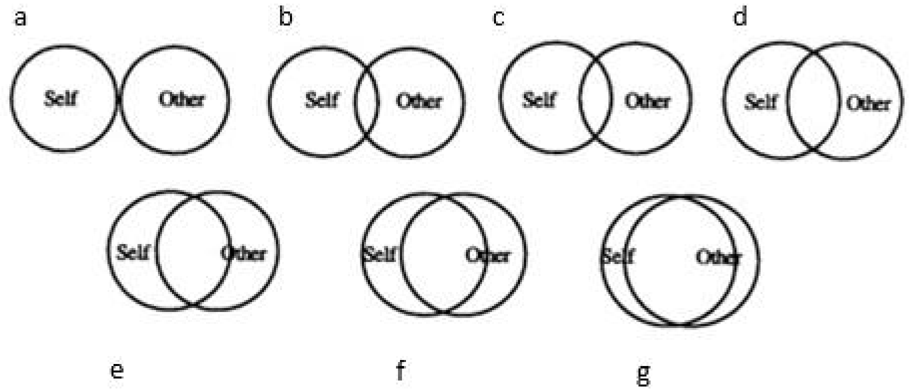

*Please chose which picture best describes your relationship with your **partner 2 (initials: ${e://Field/SecondaryInitials})***

a

b

c

d

e

f

g

Choose not to respond

Dyadic Adjustment Scale - Happiness item P2

The following represents different degrees of happiness in your relationship with **partner 2 (initials: ${e://Field/SecondaryInitials})**. Please select the choice which best describes the degree of happiness, all things considered, of your relationship with **partner 2 (initials: ${e://Field/SecondaryInitials})**.

0 Extremely unhappy

1 Fairly unhappy

2 A little unhappy

3 Happy

4 Very happy

5 Extremely happy

6 Perfect

Choose not to respond

Motives for Engaging in CNM

We're just about done! Now that we've asked you about your specific relationships, we want to ask you a few questions about why you chose to engage in a CNM relationship.

Below are some reasons that people report for engaging in consensually non-monogamous/multipartnered relationships. We would like you to think about your relationship(s) and indicate the extent to which the following statements reflect the reasons why you are engaging in a CNM/multipartnered relationship.

Reflect on the following statements about the reasons you are engaging in a CNM/multipartnered relationship and indicate the degree to which you engage in a CNM/multipartnered relationship for each reason.

	Not at all for this reason 0	A little for this reason 1	Somewhat for this reason 2	Quite a bit for this reason 3	Very much for this reason 4	Choose not to respond
Because it feels natural for me.	O	O	O	O	O	O
Because this type of relationship is reflective of my beliefs.	O	O	O	O	O	O
Because it allows me to grow as a person.	O	O	O	O	O	O
Because it allows me and my partner(s) to grow together.	O	O	O	O	O	O
Because I feel that I have the capacity to love more than one person.	O	O	O	O	O	O

	Not at all for this reason 0	A little for this reason 1	Somewhat for this reason 2	Quite a bit for this reason 3	Very much for this reason 4	Choose not to respond
Because I feel that I have the capacity to romantically connect with many people at one time.	O	O	O	O	O	O
Because I value the support that I receive in this community.	O	O	O	O	O	O
Because it fits with my lifestyle.	O	O	O	O	O	O
Because my partner and I spend a lot of time apart from one another.	O	O	O	O	O	O
Because it allows me to explore my sexual desires.	O	O	O	O	O	O

Reflect on the following statements about the reasons you are engaging in a CNM/multipartnered relationship.

	**Not at all for this reason 0**	**A little for this reason 1**	**Somewhat for this reason 2**	**Quite a bit for this reason 3**	**Very much for this reason 4**	Choose not to respond
Because it allows me to explore my sexual orientation.	○	○	○	○	○	○
Because I like having sexual variety.	○	○	○	○	○	○
Because it is pleasurable.	○	○	○	○	○	○
Because this relational style suits me.	○	○	○	○	○	○
Because I wanted to revive the passion in my primary relationship.	○	○	○	○	○	○

	**Not at all for this reason 0**	**A little for this reason 1**	**Somewhat for this reason 2**	**Quite a bit for this reason 3**	**Very much for this reason 4**	Choose not to respond
Because I enjoy seeing my partner happy in other relationships.	○	○	○	○	○	○
Because my partner wanted to.	○	○	○	○	○	○
Because I find monogamous relationships to be too constrained.	○	○	○	○	○	○
Because it allows me to meet my sexual needs.	○	○	○	○	○	○
Because it allows me to meet my emotional needs.	○	○	○	○	○	○

Reflect on the following statements about the reasons you are engaging in a CNM/multipartnered relationship.

	Not at all for this reason 0	A little for this reason 1	Somewhat for this reason 2	Quite a bit for this reason 3	Very much for this reason 4	Choose not to respond
Because it allows my partner to meet their needs.	○	○	○	○	○	○
Because I value sexual autonomy.	○	○	○	○	○	○
Because it is exciting.	○	○	○	○	○	○
Because it allows me to be myself.	○	○	○	○	○	○
Because I do not feel that I could have my needs met in a monogamous relationship.	○	○	○	○	○	○

	Not at all for this reason 0	A little for this reason 1	Somewhat for this reason 2	Quite a bit for this reason 3	Very much for this reason 4	Choose not to respond
Because I want to meet new people.	○	○	○	○	○	○
Because I want to avoid conflict with a partner.	○	○	○	○	○	○
Because I want to help fulfill my partner's fantasies.	○	○	○	○	○	○
Because I wanted to enhance my relationship with my primary partner.	○	○	○	○	○	○

Other:

FSFI q1 and q2

These are the last questions!

This questionnaire asks about your level of sexual desire. By desire, we mean interest in or wish for sexual activity.

Over the past 4 weeks, how **often** did you feel sexual desire or interest?

Almost always or always
Most times
Sometimes
A few times
Almost never or never

Over the past 4 weeks, how would you rate your **level** (degree) or sexual desire or interest?

Very high
High
Moderate
Low
Very low or none at all

Daily Survey

PP

Please complete the following questions about your **primary relationship (initials: ${e://Field/PrimaryInitials}**) today:

	**Very strong disagreement 1**	**2**	**3**	**4**	**5**	**Very strong agreement 6**	Choose not to respond
Right now my relationship with my primary partner is stable	○	○	○	○	○	○	○
Right now my relationship with my primary partner is strong	○	○	○	○	○	○	○

	**Very strong disagreement 1**	**2**	**3**	**4**	**5**	**Very strong agreement 6**	Choose not to respond
Right now my relationship with my primary partner makes me happy	○	○	○	○	○	○	○

	**Very strong disagreement 1**	**2**	**3**	**4**	**5**	**Very strong agreement 6**	Choose not to respond
Right now I am experiencing conflict with my primary partner	○	○	○	○	○	○	○
Right now I am unsure if my relationship with my primary partner will last	○	○	○	○	○	○	○
Right now my primary and I are getting on each other's nerves.	○	○	○	○	○	○	○

The following represents different degrees of happiness in your relationship. Please select the choice which best describes the degree of happiness RIGHT NOW, all things considered, of your **primary relationship (initials: ${e://Field/PrimaryInitials}).**

**Extremely unhappy 0**	**Fairly unhappy 1**	**A little unhappy 2**	**Happy 3**	**Very happy 4**	**Extremely happy 5**	**Perfect 6**	Choose not to respond

Overall, how satisfied are you today with the sexual aspect of your **primary relationship (initials: ${e://Field/PrimaryInitials})**?

**Not at all satisfied**	**A little satisfied**	**Moderately satisfied**	**Very satisfied**	**Extremely satisfied**	Choose not to respond

Today I had exciting experiences with my **primary partner (initials: ${e://Field/PrimaryInitials}).**

Not at all true 1	2	3	4	5	6	7	8	Completely True 9	Choose not to respond

My **primary partner (initials: ${e://Field/PrimaryInitials})** and I communicated well today.

Very strong disagreement 1	2	3	Moderate agreement 4	5	Very strong agreement 6	Choose not to respond

I was highly motivated to meet my **primary partner's (initials: ${e://Field/PrimaryInitials})** needs today.

Very strong disagreement 1	2	3	Moderate agreement 4	5	Very strong agreement 6	Choose not to respond

My desire for sex with my **primary partner (initials: ${e://Field/PrimaryInitials})** is strong today.

Not at all 0	1	2	3	4	Very much 5	Choose not to respond

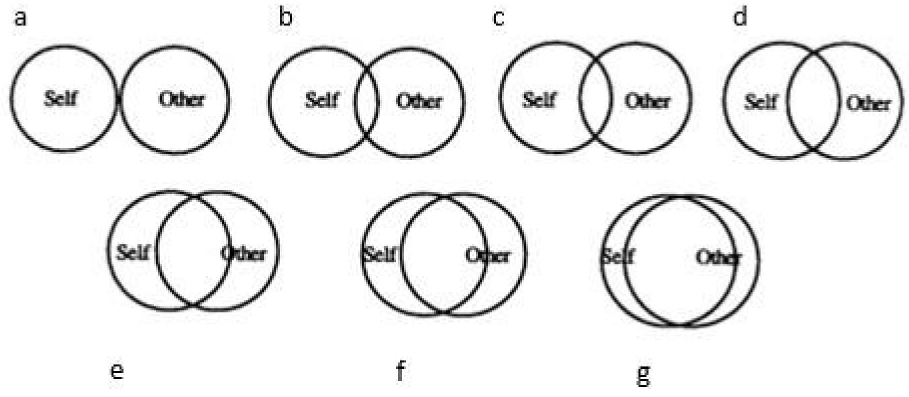

Please chose which picture best describes your relationship with your **primary partner (initials: ${e://Field/PrimaryInitials})** today.

a	b	c	d	e	f	g	Choose not

to respond

P2

The following represents different degrees of happiness in your relationship. Please select the choice which best describes the degree of happiness RIGHT NOW, all things considered, of **partner 2 (initials: ${e://Field/SecondaryInitials})**

**Extremely unhappy 0**	**Fairly unhappy 1**	**Happy 3**	**Very happy 4**	Choose not to respond

Overall, how satisfied are you today with the sexual aspect of your relationship with **partner 2 (initials: ${e://Field/SecondaryInitials})**?

**Not at all satisfied**	**A little satisfied**	**Moderately satisfied**	**Very satisfied**	**Extremely satisfied**	Choose not to respond

My desire for sex with **partner 2 (initials: ${e://Field/SecondaryInitials})** is strong today

**Not at all 0**	**1**	**2**	**3**	**4**	**Very much 5**	Choose not to respond

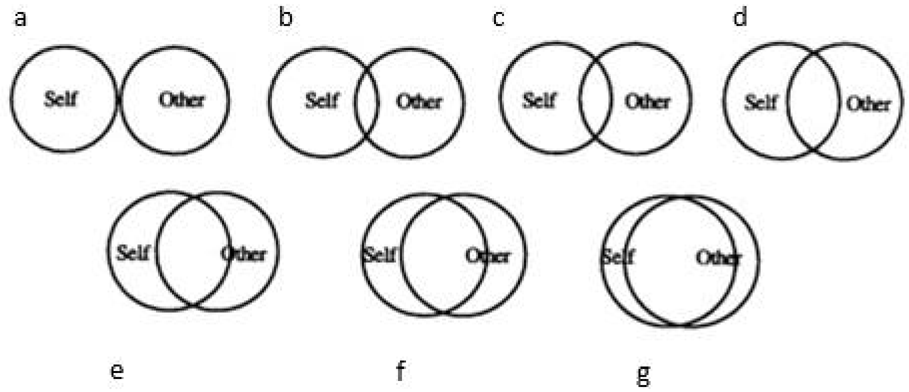

Please chose which picture best describes your relationship with **partner 2 (initials: ${e://Field/SecondaryInitials})** today.

a	b	c	d	e	f	g	Choose not to respond

Well-being

Using the 1-7 scale below, indicate your agreement with each item by placing the appropriate number on the line preceding that item. Please be open and honest in your responding.

I am satisfied with my life today.

Strongly agree 7	Agree 6	Slightly agree 5	Neither agree nor disagree 4	Slightly disagree 3	Disagree 2	Strongly disagree 1	Choose not to respond

Sexual activity

Did you engage in sexual interaction with a partner today?

Yes	No	Choose not to respond

When you engaged in sexual activity today, was it with...

My primary partner (initials: ${e://Field/PrimaryInitials})	Partner 2 (initials: ${e://Field/SecondaryInitials})	A partner not listed here	Choose not to respond

Some of the following questions ask about you last sexual encounter today.

Which sexual interaction are you reporting on today?

One with primary partner (initials: ${e://Field/PrimaryInitials})

One with partner 2 (initials: ${e://Field/SecondaryInitials})

A partner not listed here

Choose not to respond

Sexual Rejection (if NO sex day)

Today, to what extent **did YOU do any** of the following to indicate to your partner that you were not in the mood for sex? (If none of the following are applicable, please select 'not at all' for all items).

	Not at all 1	2	3	Somewhat 4	5	6	A lot 7	Choose not to respond
I laid in a position that's a hard to snuggle with	O	O	O	O	O	O	O	O
I criticized aspects of our relationship	O	O	O	O	O	O	O	O
I pretended to sleep	O	O	O	O	O	O	O	O
I was clear and direct about why I didn't want to have sex	O	O	O	O	O	O	O	O
I reassured my partner that I love them	O	O	O	O	O	O	O	O

	Not at all 1	2	3	Somewhat 4	5	6	A lot 7	Choose not to respond
I told my partner honestly the reason why I don't want to have sex	O	O	O	O	O	O	O	O
I criticized the way my partner initiated sex	O	O	O	O	O	O	O	O
I said 'no' in a direct manner	O	O	O	O	O	O	O	O
I physically turned away from my partner	O	O	O	O	O	O	O	O
I gave my partner the silent treatment	O	O	O	O	O	O	O	O

Today, to what extent **did YOU do any** of the following to indicate to your partner that you were not in the mood for sex? (If none of the following are applicable, please select

'not at all' for all items).

	Not at all 1	2	3	Somewhat 4	5	6	A lot 7	Choose not to respond
I offered to make it up to my partner in the future	○	○	○	○	○	○	○	○
I didn't reciprocate my partner's affection	○	○	○	○	○	○	○	○
I pretended not to notice that my partner was interested in sex	○	○	○	○	○	○	○	○
I offered alternate forms of physical contact (kissing, hugging, snuggling, cuddling)	○	○	○	○	○	○	○	○
I displayed frustration towards my partner	○	○	○	○	○	○	○	○

	Not at all 1	2	3	Somewhat 4	5	6	A lot 7	Choose not to respond
I was short or curt with my partner	○	○	○	○	○	○	○	○
I tried to talk with my partner instead	○	○	○	○	○	○	○	○
I reassured my partner that I am attracted to them	○	○	○	○	○	○	○	○
I was straightforward about why I rejected my partner	○	○	○	○	○	○	○	○
I was open about the reason, even if it hurt my partner's feelings	○	○	○	○	○	○	○	○

Optional: If your desired response was not listed, please elaborate.

Sexual Rejection Item set 1 - Shown if not at all = 20

You indicated that you did not engage in any of the behaviors above. Please indicate the reason why.

These behaviours were not applicable today

These behaviours were applicable but I did not engage in any of them

Other

Choose not to respond

Sexual Rejection Logic - Shown if not at all LESS than 20

How did your partner respond?

Not at all 1	2	3	Somewhat 4	5	6	A lot 7	Choose not to respond

My partner attempted to change my mind (e.g., tried to tempt or seduce me)	O	O	O	O	O	O	O	O
My partner tried not to express negative emotions	O	O	O	O	O	O	O	O
My partner let me know they still love me	O	O	O	O	O	O	O	O
My partner acted cold towards me	O	O	O	O	O	O	O	O
My partner let me know they are still attracted to me	O	O	O	O	O	O	O	O
	**Not at all 1**	**2**	**3**	**Somewhat 4**	**5**	**6**	**A lot 7**	Choose not to respond
My partner took it as an indication that I don't find them attractive	O	O	O	O	O	O	O	O
My partner thought something is wrong in the relationship	O	O	O	O	O	O	O	O
My partner tried to make me feel guilty	O	O	O	O	O	O	O	O
My partner asked if there is anything they can do to get me in the mood	O	O	O	O	O	O	O	O
My partner was upset or sad	O	O	O	O	O	O	O	O

How did your partner respond?

	Not at all 1	2	3	Somewhat 4	5	6	A lot 7	Choose not to respond
My partner hugged/kissed me	O	O	O	O	O	O	O	O

	Not at all 1	2	3	Somewhat 4	5	6	A lot 7	Choose not to respond
My partner continued trying to convince me to have sex	○	○	○	○	○	○	○	○
My partner acted quiet	○	○	○	○	○	○	○	○
My partner expressed anger at me	○	○	○	○	○	○	○	○
My partner was understanding and accepting	○	○	○	○	○	○	○	○

	Not at all 1	2	3	Somewhat 4	5	6	A lot 7	Choose not to respond
My partner was offended/hurt	○	○	○	○	○	○	○	○
My partner ignored me	○	○	○	○	○	○	○	○
My partner tried initiating sex with me again	○	○	○	○	○	○	○	○
My partner tried to get me in the mood	○	○	○	○	○	○	○	○
My partner accused me of being selfish	○	○	○	○	○	○	○	○

Optional: If your desired response was not listed, please elaborate.

Non sex days open ended

Is there anything else about your relationship(s) that you would like to share with us today?

Sexual Motives (if sex day)

Please respond to the following items about your sexual motivations.

	**Not at all for this reason**	**A little for this reason**	**Somewhat for this reason**	**Quite a bit for this reason**	**Very much for this reason**	Choose not to respond
Because I want to enjoy the physical sensations	O	O	O	O	O	O
Because I think it feels good	O	O	O	O	O	O
Because my sexual desire is high	O	O	O	O	O	O
Because I enjoy being sexual	O	O	O	O	O	O
Because I wanted a fun experience	O	O	O	O	O	O
	**Not at all for this reason**	**A little for this reason**	**Somewhat for this reason**	**Quite a bit for this reason**	**Very much for this reason**	Choose not to respond

	Not at all for this reason	A little for this reason	Somewhat for this reason	Quite a bit for this reason	Very much for this reason	Choose not to respond
Because I think sex makes me feel better about myself	○	○	○	○	○	○
Because I want to show how good I am in bed	○	○	○	○	○	○
Because the proposition makes me feel more attractive	○	○	○	○	○	○
Because I want to feel more powerful or dominant	○	○	○	○	○	○
Because I wanted to show that i was capable of performing	○	○	○	○	○	○

	Not at all for this reason	A little for this reason	Somewhat for this reason	Quite a bit for this reason	Very much for this reason	Choose not to respond
Because I expect a satisfying deep connection with my partner(s) during sex	○	○	○	○	○	○
Because I want sex to be a celebration of the feelings between my partner(s) and me	○	○	○	○	○	○
Because I value how sex can being me closer to another person/other people	○	○	○	○	○	○

Please respond to the following items about your sexual motivations.

	Not at all for this reason	A little for this reason	Somewhat for this reason	Quite a bit for this reason	Very much for this reason	Choose not to respond
For the pleasure of sharing a special and intimate experience	○	○	○	○	○	○

	Not at all for this reason	A little for this reason	Somewhat for this reason	Quite a bit for this reason	Very much for this reason	Choose not to respond
Because I want to enjoy the closeness of being physically joined with my partner(s)	O	O	O	O	O	O
Because I think saying no will start a conflict with my partner(s)	O	O	O	O	O	O
Because I feel anxious or guilty if I don't go along	O	O	O	O	O	O

	Not at all for this reason	A little for this reason	Somewhat for this reason	Quite a bit for this reason	Very much for this reason	Choose not to respond
Because I worry my partner(s) might leave or reject me if I don't have sex	O	O	O	O	O	O
Because I feel pressured by my partner(s) to have sex	O	O	O	O	O	O
Because I worry I will be punished or neglected by my partner(s) if i don't	O	O	O	O	O	O

Sexual Need Satisfaction (if sex day)

Please read and rate the following items...

	Strongly disagree 1	2	3	4	5	6	Strongly agree 7	Choose not to respond
My sexual needs were met during this sexual interaction...	O	O	O	O	O	O	O	O
My emotional needs were met during this sexual interaction...	O	O	O	O	O	O	O	O

During the sexual interaction I felt...

	Strongly disagree 1	2	3	4	5	6	Strongly agree 7	Choose not to respond
Choiceful	○	○	○	○	○	○	○	○
Competent	○	○	○	○	○	○	○	○
Connected to my partner	○	○	○	○	○	○	○	○
A lot of closeness and intimacy	○	○	○	○	○	○	○	○
My feelings and wishes were respected	○	○	○	○	○	○	○	○
Inadequate	○	○	○	○	○	○	○	○

How pleasurable was your sexual experience today?

Very pleasurable	Somewhat pleasurable	Not very pleasurable	Not at all pleasurable	Choose not to respond

Sex days open ended

Is there anything else you would like to tell us about today's sexual encounter?
