# Supplementary material for: A dyadic examination of self-determined sexual motives, need fulfillment, and relational outcomes among consensually non-monogamous partners
Source: PLoS One. 2021 Feb 16;16(2):e0247001. doi: 10.1371/journal.pone.0247001 (PMC7886188; doi:10.1371/journal.pone.0247001)
Supplement: S2 File — (DOCX) [file pone.0247001.s002.docx]

Online Supplement 2

| Model | Distinguishability test | Outcome |
| --- | --- | --- |
| Primary Partner Models |  |  |
| Rel. Sat. 🡨 Need. Sat. | *X*^2^ (4) =.777, *p* = .94 | Indistinguishable supported |
| Sex. Sat. 🡨 Motives | *X*^2^ (4) = 2.432, *p* = .65 | Indistinguishable supported |
| Need. Sat. 🡨 Motives | *X*^2^ (4) = 2.717, *p* = .61 | Indistinguishable supported |
| Secondary Partner Models |  |  |
| Rel. Sat. 🡨Need. Sat. | *X*^2^ (4) = 3.87, *p* = .42 | Indistinguishable supported |
| Sex. Sat. 🡨 Motives | *X*^2^ (4) = 5.008, *p* = .29 | Indistinguishable supported |
| Need. Sat. 🡨 Motives | *X*^2^ (4) =.276, *p* = .92 | Indistinguishable supported |

Summary of Distinguishability Tests for Intake (day 0) Models

| Model | Distinguishability test | Outcome |
| --- | --- | --- |
| Sex. Sat. 🡨 Motives | *X*^2^ (7) = 9.968, *p* = .17 | Indistinguishable supported |
| Rel. Sat. 🡨 Motives | *X*^2^ (7) =20.218, *p* = .002 | Distinguishable supported |
| Need. Sat. 🡨 Motives | *X*^2^ (7) = 7.97, *p* = .24 | Indistinguishable supported |
| Rel. Sat. 🡨 Need. Sat. | *X*^2^ (7) = 15.935, *p* = .01 | Distinguishable supported |
| Sex. Sat. 🡨 Need. Sat. | *X*^2^ (7) = 27.046, *p* < .001 | Distinguishable supported |

Summary of Distinguishability Tests for Daily Diary Models

Summary of Distinguishability Tests for 3-Month Follow Up Models

| Model | Distinguishability test | Outcome |
| --- | --- | --- |
| Actor Rel. Sat. 🡨 Motives | *X*^2^ (4) = 0.598, *p* = .96 | Indistinguishable supported |
| Partner Rel. Sat. 🡨 Motives | *X*^2^ (4) = 1.473, *p* = .83 | Indistinguishable supported |
| Actor Sex. Sat. 🡨 Motives | *X*^2^ (4) = 10.714, *p* = .03 | Distinguishable supported |
| Partner Sex. Sat. 🡨 Motives | *X*^2^ (4) = 9.368, *p* =. 052 | Indistinguishable supported |
| Need. Sat. 🡨 Motives | *X*^2^ (4) = 9.281, *p* = .098 | Indistinguishable supported |

*Table 5a.*

Daily associations between actor and partner sexual motives and sexual need fulfillment and daily relationship satisfaction, sexual satisfaction, and sexual need fulfillment. Results of distinguishable models (by gender)

|  | | Relationship Satisfaction | | | Sexual Satisfaction | | | Sexual Need Fulfillment | |
| --- | --- | --- | --- | --- | --- | --- | --- | --- | --- |
|  | *b* (SE) | | *t* | *b* (SE) | | *t* | *b* (SE) | | *t* |
| Actor Motives | .01 | | .12 | ----- | | ----- | ----- | | ----- |
| Partner Motives | .07 | | 1.15 | ----- | | ----- | ----- | | ----- |
| Relationship Length | .01 | | 1.86 | ----- | | ----- | ----- | | ----- |
| Gender | .45 | | 2.12* | ----- | | ----- | ----- | | ----- |
| Gender* Actor Motives | .01 | | .38 | ----- | | ----- | ----- | | ----- |
| Gender* Partner Motives | -.03 | | -.93 | ----- | | ----- | ----- | | ----- |
| Actor Sexual  Need Fulfillment | .05 | | .26 | .46 | | 1.74 | ----- | | ----- |
| Partner Sexual Need Fulfillment | .04 | | .17 | .03 | | .11 | ----- | | ----- |
| Relationship Length | .01 | | 2.39* | -.03 | | -3.57*** | ----- | | ----- |
| Gender | 1.31 | | 2.58* | -2.633 | | -2.85** | ----- | | ----- |
| Gender*Actor Sexual  Need Fulfillment | .03 | | .21 | -.10 | | -.55 | ----- | | ----- |
| Gender*Partner Sexual  Need Fulfillment | -.00 | | -.04 | .05 | | .29 | ----- | | ----- |

Note: *b* values are unstandardized coefficients; degrees of freedom ranged from 396.52 to 428.25

**p*<.05, ** *p*<.01, *** *p*<.001

*Table 7a.* Sexual motives predicting relationship satisfaction, sexual satisfaction, and sexual need fulfillment at a three-month follow-up. Results of distinguishable models (by gender)

|  | Relationship Satisfaction | | Sexual Satisfaction | | Sexual Need Fulfillment | |
| --- | --- | --- | --- | --- | --- | --- |
|  | *b* (SE) | *t* | *b* (SE) | *t* | *b* (SE) | *t* |
| Actor Motives | - | - | .08 | 1.03 | - | - |
| Relationship Length | - | - | -.08 | -2.41* | - | - |
| Gender | - | - | -.64 | -2.37 | - | - |
| A. Motives*Gender | - | - | .06 | 1.11 | - | - |
| Partner Motives | - | - | -.02 | -.18 | - | - |
| Relationship Length | - | - | -.03 | -.84 | - | - |
| Gender | - | - | .51 | 1.87 | - | - |
| P. Motives*Gender | - | - | .00 | .07 | - | - |

Note: *b* values are unstandardized coefficients. All models also include relationship length as a covariate.

**p*<.05, ** *p*<.01, *** *p*<.001
