# Supplementary material for: A dyadic examination of self-determined sexual motives, need fulfillment, and relational outcomes among consensually non-monogamous partners
Source: PLoS One. 2021 Feb 16;16(2):e0247001. doi: 10.1371/journal.pone.0247001 (PMC7886188; doi:10.1371/journal.pone.0247001)
Supplement: S3 File — (DOCX) [file pone.0247001.s003.docx]

Online Supplement 3: Tables and Results Without Controlling for Relationship Length

*Table 3.* Associations between actor and partner sexual motives and relationship satisfaction, sexual satisfaction, and sexual need fulfillment with the first partner

|  | Relationship Satisfaction | | Sexual Satisfaction | | Sexual Need Fulfillment | |
| --- | --- | --- | --- | --- | --- | --- |
|  | *b* (SE) | *t* | *b* (SE) | *t* | *b* (SE) | *t* |
| Actor Motives | .03 (.02) | 1.39 | 1.07 (.15) | 7.04*** | .09 (.02) | 5.10*** |
| Partner Motives | .04 (.02) | 1.67 | 0.20 (.15) | 1.31 | .04 (.02) | 2.25* |
| Actor Sexual  Need Fulfillment | .51 (.09) | 5.96*** | 6.00 (.81) | 7.38*** | ----- | ----- |
| Partner Sexual Need Fulfillment | .13 (.09) | 1.49 | -.32 (.80) | -0.39 | ----- | ----- |

Note: *b* values are unstandardized coefficients

**p*<.05, ** *p*<.01, *** *p*<.001

*Table 4.* Associations between actor and partner sexual motives and sexual need fulfillment with the second partner, and relationship satisfaction and sexual satisfaction with the first partner

|  | Relationship Satisfaction | | Sexual Satisfaction | | Sexual Need Fulfillment | |
| --- | --- | --- | --- | --- | --- | --- |
|  | *b* (SE) | *t* | *b* (SE) | *t* | *b* (SE) | *t* |
| Actor Motives | .04 (.03) | 1.37 | .22 (.23) | 0.97 | .02 (.03) | 0.87 |
| Partner Motives | .01 (.03) | 0.46 | -0.03 (.23) | -0.12 | .02 (.03) | 0.67 |
| Actor Sexual  Need Fulfillment | .10 (.11) | 0.97 | 1.37 (.86) | 1.59 | ----- | ----- |
| Partner Sexual Need Fulfillment | -.12 (.11) | -1.14 | -2.53 (.87) | -2.92** | ----- | ----- |

Note: *b* values are unstandardized coefficients

**p*<.05, ** *p*<.01, *** *p*<.001

*Table 5.* Daily associations between actor and partner sexual motives and sexual need fulfillment and daily relationship satisfaction, sexual satisfaction, and sexual need fulfillment

|  | Relationship Satisfaction | | Sexual Satisfaction | | Sexual Need Fulfillment | |
| --- | --- | --- | --- | --- | --- | --- |
|  | *b* (SE) | *t* | *b* (SE) | *t* | *b* (SE) | *t* |
| Actor Motives | .01 (.01) | 1.27 | .07 (.02) | 4.67*** | .13 (.01) | 8.70*** |
| Partner Motives | .004 (.01) | 0.35 | .03 (.02) | 2.03* | .03 (.01) | 2.23* |
| Actor Sexual  Need Fulfillment | .06 (.04) | 1.68 | .28 (.05) | 5.15*** | ----- | ----- |
| Partner Sexual Need Fulfillment | .001 (.04) | 0.03 | .06 (.05) | 1.07 | ----- | ----- |

Note: *b* values are unstandardized coefficients; degrees of freedom ranged from 401.19 to 428.25

**p*<.05, ** *p*<.01, *** *p*<.001

*Table 6.* Daily associations between actor and partner sexual motives, sexual need fulfillment, and sexual satisfaction (with participants’ first partner) by partner type

|  | Sexual Need Fulfillment | | Sexual Satisfaction | |
| --- | --- | --- | --- | --- |
|  | *b* (SE) | *t* | *b* (SE) | *t* |
| Actor Motives FP | .10 (.01) | 7.74*** | .09 (.02) | 5.20*** |
| Actor Motives AP | .18 (.02) | 8.74*** | .02 (.03) | 0.76 |
| Partner Motives FP | .04 (.01) | 2.74** | .01 (.02) | 0.82 |
| Partner Motives AP | .01 (.02) | 0.32 | .08 (.03) | 2.76** |
| Actor Sexual Need Fulfillment FP | ----- | ----- | .34 (.07) | 4.88*** |
| Actor Sexual Need Fulfillment AP | ----- | ----- | .08 (.10) | 0.79 |
| Partner Sexual Need Fulfillment FP | ----- | ----- | .02 (.07) | 0.23 |
| Partner Sexual Need Fulfillment AP | ----- | ----- | .12 (.10) | 1.20 |

Note: *b* values are unstandardized coefficients

**p*<.05, ** *p*<.01, *** *p*<.001

FP: First partner

AP: Another partner

*Table 7.* Sexual motives predicting relationship satisfaction, sexual satisfaction, and sexual need fulfillment at a three-month follow-up.

|  | Relationship Satisfaction | | Sexual Satisfaction | | Sexual Need Fulfillment | |
| --- | --- | --- | --- | --- | --- | --- |
|  | *b* (SE) | *t* | *b* (SE) | *t* | *b* (SE) | *t* |
| Actor Motives | .31 (11) | 2.90** | .06 (.03) | 2.42* | .20 (.02) | 11.67*** |
| Partner Motives | -.13 (11) | -1.25 | -.002 (.03) | -0.06 | .14 (.02) | 7.24*** |

Note: *b* values are unstandardized coefficients

**p*<.05, ** *p*<.01, *** *p*<.001
